# Mll4 in skeletal muscle fibers maintains muscle stem cells

**DOI:** 10.1186/s13395-024-00369-9

**Published:** 2024-12-23

**Authors:** Yea-Eun Kim, Sang-Hyeon Hann, Young-Woo Jo, Kyusang Yoo, Ji-Hoon Kim, Jae W. Lee, Young-Yun Kong

**Affiliations:** 1 School of Biological Sciences, Seoul National University, Seoul, 08826 Republic of Korea; 2 Molecular Recognition Research Center, Korea Institute of Science and Technology, Seoul, 02792 Republic of Korea; 3 Department of Biological Sciences, University at Buffalo, Buffalo, NY 142604 USA

**Keywords:** Skeletal muscle, Myofiber, Muscle stem cells, Myeloid/lymphoid or mixed-lineage leukemia 4 (Mll4), Exercise, Notch signaling

## Abstract

**Background:**

Muscle stem cells (MuSCs) undergo numerous state transitions throughout life, which are critical for supporting normal muscle growth and regeneration. Epigenetic modifications in skeletal muscle play a significant role in influencing the niche and cellular states of MuSCs. Mixed-lineage leukemia 4 (Mll4) is a histone methyltransferase critical for activating the transcription of various target genes and is highly expressed in skeletal muscle. This raises the question of whether Mll4 has a regulatory function in modulating the state transitions of MuSCs, warranting further investigation.

**Methods:**

To assess if myofiber-expressed Mll4, a histone methyltransferase, contributes to the maintenance of MuSCs, we crossed *MCK*^*Cre/+*^ or *HSA*^*MerCreMer/+*^ mice to *Mll4*^*f/f*^ mice to generate myofiber-specific *Mll4*-deleted mice. Investigations were conducted using 8-week-old and 4-week-old *MCK*^*Cre/+*^;*Mll4*^*f/f*^ mice, and adult *HSA*^*MerCreMer/+*^;*Mll4*^*f/f*^ mice between the ages of 3 months and 6 months.

**Results:**

During postnatal myogenesis, *Mll4* deleted muscles were observed with increased number of cycling MuSCs that proceeded to a differentiation state, leading to MuSC deprivation. This phenomenon occurred independently of gender. When *Mll4* was ablated in adult muscles using the inducible method, adult MuSCs lost their quiescence and differentiated into myoblasts, also causing the depletion of MuSCs. Such roles of *Mll4* in myofibers coincided with decreased expression levels of distinct Notch ligands: *Jag1* and *Dll1* in pubertal and *Jag2* and *Dll4* in adult muscles.

**Conclusions:**

Our study suggests that *Mll4* is crucial for maintaining MuSCs in both pubertal and adult muscles, which may be accomplished through the modulation of distinct Notch ligand expressions in myofibers. These findings offer new insights into the role of myofiber-expressed Mll4 as a master regulator of MuSCs, highlighting its significance not only in developmental myogenesis but also in adult muscle, irrespective of sex.

**Supplementary Information:**

The online version contains supplementary material available at 10.1186/s13395-024-00369-9.

## Background

Muscle stem cells (MuSCs) are resident stem cells of skeletal muscle that contribute to muscle development, growth, and regeneration. They actively proliferate and differentiate into myocytes to contribute to myonuclei accretion and muscle growth [1, 2]. In the adult stage, MuSCs enter a quiescent state and remain as reserve stem cells [3, 4]. Upon injury, MuSCs become activated, providing myogenic cells to repair the muscle tissue. During muscle development and regenerative myogenesis, the MuSC niche regulates the dynamic transitions of MuSCs, including their activation, proliferation, differentiation, and self-renewal.

Myofiber is an important cellular component of the MuSC niche. Unlike other cells that compose the MuSC niche, myofibers are in direct contact with MuSCs [5]. This enables them to regulate the MuSC state through both paracrine and contact-dependent juxtacrine signaling [6]. As paracrine signaling, myofibers secrete Wnt-4 to repress aberrant activation and maintain MuSC quiescence in adult homeostatic muscle [7]. FGF6 is another paracrine factor produced in myofibers to promote MuSC expansion during both developmental and regenerative myogenesis [8, 9]. Myofibers also provide juxtacrine signals such as N-cadherin and M-cadherin to maintain MuSCs. These cell adhesion molecules are expressed at myofiber sites that are in direct contact with MuSCs to repress stem cell activation and maintain MuSC quiescence in adult muscles [10].

Among the various molecular signals derived from myofibers, Notch signaling plays a particularly crucial role in the maintenance of the stem cell pool and the regulation of cell fate decisions in MuSCs. In mammals, Notch signal-sending cells express Notch ligands (Dll1, 4 and Jag1, 2) and signal-receiving cells express Notch receptors (Notch1-4) [11, 12]. Myofibers activate Notch signaling at different developmental stages primarily to generate a quiescent population of myogenic progenitor cells [2, 13]. Several Notch ligands are expressed in myofibers to facilitate diverse Notch signaling to regulate the MuSC niche effectively at different developmental stages. Dll1, a Notch ligand mainly expressed in pubertal myofibers, interacts with the activated MuSCs to promote self-renewal, which is crucial for maintaining MuSCs [14–16]. In adult myofibers, Dll4 represses MuSC cell cycle entry, thus retaining the quiescent state of MuSCs [17, 18]. While various studies have indicated that Notch signaling originating from myofibers has a role in regulating the state of MuSCs, there is limited understanding of whether there is a regulator present in myofibers that coordinates the expression of different Notch ligands among different postnatal periods such as puberty and adulthood.

Mixed-lineage leukemia 4 (MLL4; also known as Kmt2d), a major H3K4 mono- and di-methyltransferase, is an essential histone modification enzyme for enhancer activation [19–21]. H3K4me1 marking by MLL4 is required for H3K27 acetylation and the recruitment of cell type-specific transcription factors [19–22]. Deletion of *Mll4* results in the disturbance of H3K4me1 and H3K27ac on active enhancers, leading to defects in transcribing both newly activated genes, as well as genes that were already being expressed [19–22].

A recent study addressed that *Mll4* depletion in myofibers turns off the slow type I fiber-specific genes, leading to fiber-type transition [23]. Myofibers are classified into two primary types: Type 1 (slow-twitch) and Type 2 (fast-twitch). Type 1 fibers rely on oxidative metabolism, which endows them with endurance capabilities. In contrast, Type 2 fibers utilize glycolytic pathways to generate rapid force [24]. This diversity in myofiber types contributes to muscle performance and adaptability to various stimuli [25]. Liu [23] reported that endurance exercise capacity was reduced in the Mll4-KO mice due to a slow-to-fast fiber-type shift. However, this study exclusively utilized male mice and focused solely on the function of Mll4 in developing myofibers. Building on these findings, our research aims to further investigate the role of Mll4 in myofibers across both sexes and various developmental stages.

In this paper, we report that MLL4 in myofiber is required to maintain MuSCs during both the pubertal and adult stages, regardless of gender. Mouse models with myofiber-specific deletion of *Mll4* exhibited reduced myofiber length, fewer myonuclei, and MuSC depletion. When *Mll4* was ablated at the adult stage using an inducible method, MuSCs underwent differentiation into myoblasts, either with or without entering the cell cycle, leading to a depletion of adult MuSCs. Furthermore, the expression of specific Notch ligands at both pubertal and adult stages was significantly reduced in *Mll4*-knockout myofibers. Together, our data suggest the importance of MLL4 in skeletal muscle fibers for maintaining the MuSC number, which might be achieved by affecting Notch ligand expression, in different postnatal periods.

## Methods

### Animals

*MCK*^*Cre/+*^ (stock 006475), *HSA*^*MerCreMer/+*^ (stock 031934), and *Mll4*^*f/f*^ (stock 032152) mice were acquired from The Jackson Laboratory (Bar Harbor, ME, USA). The mice were backcrossed to C57BL/6 mice at least 6 times. To generate mice with myofiber-specific deletion of *Mll4*, *Mll4*^*f/f*^ mice were crossed with *MCK*^*Cre/+*^ (*MCK*^*Cre/+*^; *Mll4*^*f/f*^*– Mll4*^*ΔMCK*^*–*). To develop myofiber-specific *Mll4* conditional knockout mice using tamoxifen-inducible Cre, *Mll4*^*f/f*^ mice were crossed with *HSA*^*MerCreMer/+*^ (*HSA*^*MerCreMer/+*^; *Mll4*^*f/f*^*– Mll4*^*ΔHSA*^*–*). Following a breeding strategy from The Jackson Laboratory (Bar Harbor, ME, USA), we bred heterozygous *Mll4*^*f/+*^ females with Cre-recombinase to homozygous *Mll4*^*f/f*^ males. Both male and female mice were used in the experiments. Mll4^ΔHSA^ mice used for experiments were adults, between 3 and 6 months of age. Control littermates lacking Cre-recombinase (Mll4^WT^) were utilized for analysis. All mouse lines were housed under controlled conditions that were specific pathogen-free and handled according to the guidelines of the Seoul National University Institutional Animal Care and Use Committee (Protocol number: SNU-240103-3).

### Animal procedures

Tamoxifen (Sigma-Aldrich) was dissolved in corn oil at a concentration of 20 mg/ml. For tamoxifen-induced Cre recombination in Mll4^ΔHSA^ mice, both control and experimental mice were administered tamoxifen at a concentration of 150 mg/kg of mouse per day for five continuous days by intraperitoneal injection. For detection of cell cycle entry, 5-ethynyl-2’-deoxyuridine (EdU; Thermo Fisher Scientific) dissolved in sterile phosphate-buffered saline (PBS) was injected at a concentration of 40 mg/kg of mouse intraperitoneally daily.

### Muscle injury

For BaCl_2_ muscle injury, mice were anesthetized with 2.4% 2, 2, 2-Tribromoethanol (Avertin; Sigma-Aldrich) in PBS (240 mg/kg of mouse) and injected with 50 µl 1.2% BaCl_2_ in saline (Sigma-Aldrich) to the tibialis anterior (TA) muscles. At 10 days after the injury, TA muscles were dissected, frozen in optimal cutting temperature compound (O.C.T.; Sakura Finetek) with liquid nitrogen, and stored at -80 °C until analysis.

### Measurement of CSA distribution

Laminin-stained section was imaged with EVOS FL Auto 2 (Thermo Fisher Scientific) with the same laser setting, exposure, and magnification. To measure CSA, semiautomatic muscle analysis using segmentation of histology (SMASH) was used with a segmentation filter (CSA between 200 µm2 and 6000 µm2; eccentricity ≤ 0.95; convexity ≥ 0.80). For injured muscles, CSA of regenerating myofibers with centralized nuclei was analyzed. The segmentation filter was adjusted as follows: CSA between 150 µm2 and 6000 µm2; eccentricity ≤ 0.95; convexity ≥ 0.80.

### Single myofiber isolation

Single myofiber isolation was performed according to a previously reported protocol with modifications [26]. Dissected hindlimb extensor digitorum longus (EDL) muscles were enzymatically digested in Dulbecco’s modified Eagle’s medium (DMEM; Hyclone) containing 2.5% HEPES (Sigma-Aldrich) and collagenase II (800 units/mL, Worthington) at 37℃ for 60 min. Digested muscles were blocked in Dulbecco’s modified Eagle’s medium and 10% horse serum (Hyclone). The single myofibers were released by gentle trituration. Undamaged and noncontracted single myofibers were then washed with PBS several times and collected for immunocytochemistry and RNA extraction. This protocol yields a single myofiber with MuSCs attached. Consequently, RNA extracted from isolated myofibers may include a minimal fraction of MuSC-derived RNA.

### Muscle stem cell (MuSC) isolation

Isolation of MuSC was performed according to a previously reported protocol with modifications [27]. Limb muscles were dissected and mechanically dissociated in DMEM containing 10% horse serum, collagenase II (800 units/mL), and dispase (1.1 units/mL, Thermo Fisher Scientific) at 37℃ for 40 min. Digested suspensions were subsequently triturated by sterilized syringes with 20G 1/2 needle (BD Biosciences) and washed with DMEM to harvest mononuclear cells. Mononuclear cells were stained with anti-Sca-1-Pacific blue (Biolegend), anti-CD31-APC (Biolegend), anti-CD45–APC (Biolegend), and anti-Vcam1-Biotin (BD Biosciences). PE-Cy7- Streptavidin (Biolegend) was used as a secondary reagent. To exclude dead cells, 7-AAD (Sigma-Aldrich) was used. Stained cells were analyzed and Vcam1^+^Sca1^−^ 7-AAD^–^CD31– CD45– MuSCs were isolated using FACS Aria III cell sorter (BD Biosciences) with 4-way-purity precision. FACS gating strategy was referred to the previously reported protocol [27]. Isotype control density plots were used as a reference for positive gating. Freshly isolated MuSCs were attached to a slide glass by cytospin for immunocytochemistry, or collected for RNA and protein extraction.

### Immunohistochemistry

Freshly dissected TA or Soleus muscles were embedded in O.C.T., snap-frozen in liquid nitrogen, and stored at -80 °C prior to sectioning. Cross-sectional 7 μm-thick sections were obtained from the embedded muscles using a cryostat. For myosin heavy chain (MyHC) staining, unfixed muscle sections were incubated overnight at 4 °C with mouse anti-MyHC type 1 (DSHB, BA-D5, 1:10) or mouse anti-MyHC type 2x (DSHB, 6 H-1, 1:5) in addition to rat anti-laminin (Abcam, ab11576, 1:1000 dilution) in 3% BSA blocking buffer. After washes in PBS, sections were incubated for 1 h with 1:500 dilution of Alexa Fluor 488-goat anti-mouse MIgG2b (Invitrogen), or Alexa Fluor 488-conjugated anti-mouse IgM (Invitrogen), and Alexa Fluor 594-conjugated anti-rat IgG (Invitrogen). For staining myoblasts, sections were fixed in 4% paraformaldehyde (PFA) for 10 min, and washed in PBS. Antigen retrieval was then performed in citrate buffer (10 mM citric acid, pH 6) at 95 °C 15 min. The sections were blocked by mouse Ig blocking reagent and blocking buffer from M.O.M. Kit (Vector Laboratories), according to the manufacturer’s protocol. Then, the sections were incubated with primary antibodies in the blocking buffer at 4 °C overnight. The primary antibodies used include mouse anti-Pax7 (1:100, DSHB), rabbit anti-Ki67 (1:500, Sigma-Aldrich), mouse anti-MyoG (1:100, DSHB), rabbit anti-Dystrophin (1:500, Abcam), rabbit anti-Laminin (1:500, Sigma-Aldrich), and rabbit anti-cleaved Caspase-3 (1:200, Cell Signaling Technologies). After washing the sections with PBS, the sections were stained with secondary antibodies for 1 h at RT, washed, and mounted. The secondary antibodies were used at a concentration of 1:400 and include goat anti-Rabbit IgG-Alexa Fluor 488 (Thermo Fisher Scientific), goat anti-Rabbit IgG-Alexa Fluor 594 (Thermo Fisher Scientific), and goat anti-Mouse IgG-Alexa Fluor 594 (Thermo Fisher Scientific). Hoechst 33,342 (1:2,000, Thermo Fisher Scientific) was used to visualize nuclei. For EdU staining, we used the Click-iT EdU Alexa Fluor 488 Imaging Kit (Thermo Fisher Scientific) following the manufacturer’s protocol before the blocking step. The number of each cell type and myofibers was counted in the total TA or Soleus area, and representative images were selected in the same region of the section used in the cell counting. Imaging was conducted with EVOS FL Auto 2 (Thermo Fisher Scientific).

## Immunocytochemistry

For isolated MuSC staining, freshly isolated MuSCs were fixed immediately following FACS isolation. The total time required to fix the MuSCs after enzymatic dissociation and isolation was less than 3 h. Fixation was done using 4% PFA for 10 min at room temperature (RT), quenched in 0.1 M glycine in PBS for 10 min at RT, and blocked for 1 h at RT by blocking buffer (5% goat serum and 5% bovine serum albumin in PBS/0.4% Triton X-100). Then, the myofibers were incubated with mouse anti-Pax7 (1:100, DSHB) in the blocking buffer at 4 °C overnight. After washing the myofibers three times with PBS/0.1% Triton X-100, the myofibers were stained with goat anti-Mouse IgG-Alexa Fluor 594 (1:400, Thermo Fisher Scientific) and Hoechst 33,342 (1:5,000, Thermo Fisher Scientific) for 1 h at RT, washed and mounted on slide glass. Imaging was conducted with EVOS FL Auto 2 (Thermo Fisher Scientific). For isolated MuSCs staining, freshly isolated MuSCs were attached to a slide glass by cytospin and fixed by 4% PFA for 10 min at RT. The fixed MuSCs were washed with PBS/0.4% Triton X-100 several times and blocked with blocking buffer (5% goat serum and 5% bovine serum albumin in PBS/0.4% Triton X-100) for 1 h at RT and incubated with mouse anti-Pax7 (1:100, DSHB) and rabbit anti-MyoD (1:200, Santa Cruz) overnight at 4 °C. The slides were washed with PBS/0.1% Triton X-100 several times and incubated with goat anti-Mouse IgG-Alexa Fluor 594 (1:400, Thermo Fisher Scientific) and goat anti-Rabbit IgG-Alexa Fluor 488 (1:400, Thermo Fisher Scientific). For EdU staining, we used the Click-iT EdU Alexa Fluor 647 Imaging Kit (Thermo Fisher Scientific) following the manufacturer’s protocol before the blocking step. The slides were counterstained with Hoechst 33,342 (Thermo Fisher Scientific) and mounted. Imaging was conducted with EVOS FL Auto 2 (Thermo Fisher Scientific).

### Four limb grip strength measurement

Grip strength was assessed by using a grip strength test meter (grip strength test BIO-GS3, Bioseb). Mice were allowed to grasp a grid attached to the tester with 4 limbs and were manually pulled in a horizontal direction by the tip of the tail. The test was performed 5 times with 10 min of resting between each measurement. The average of the top 3 result value (N, Newton) was normalized to body weight (g) (N/g). All experiments were performed in a blinded fashion.

### Chronic exercise training and endurance running test

Randomized mice were pre-acclimated to the treadmill (DJ2-242, Dual Treadmill, Daejeon, Korea) before training. The scheme consists of exploration (0 m/min for 5 min), and subsequent running (5 m/min for 5 min, 10 m/min for 5 min, 15 m/min for 5 min). After 3 days of acclimation, mice were subjected to chronic exercise training for 5 weeks, 5 days per week with a protocol of 5 m/min for 5 min, 10 m/min for 5 min, 15 m/min for 30 min. To test endurance running capacity, mice were allowed to run until exhaustion with speed set to 10 m/min for 30 min and incremented by 2 m/min every 20 min with no inclination. Exhaustion was defined as the condition where mice remained stationary at the end of the treadmill for more than 10 s despite mechanical stimulation. All experiments were performed in a blinded fashion.

### RNA extraction and quantitative real-time polymerase chain (qRT-PCR)

Total RNA was extracted from freshly isolated myofibers (100 myofibers per mouse) and MuSCs (5,000–10,000 cells per mouse) using a TRIzol Reagent (Life Technologies) and analyzed by qRT-PCR. First-strand complementary DNA was synthesized from 1 µg of RNA using ReverTra Ace (Toyobo) containing random oligomer according to the manufacturer’s instructions. qRT-PCR (Qiagen) was performed with SYBR Green technology (SYBR Premix Ex Taq, Qiagen) using specific primers against indicated genes. Relative mRNA levels were determined using the 2^−ΔΔCt^ method and normalized to *Gapdh*. Primers are listed in Supplementary Table 1.

### Statistical analysis

Sample size determination was based on anticipated variability and effect size that was observed in the investigator’s lab for similar experiments. For quantification, individuals performing the counts were blinded to sample identity and randomized. All statistical analyses were performed using GraphPad Prism 9 (GraphPad Software). For comparison of significant differences in multiple groups for normally distributed data, statistical analysis was performed by one-way or two-way ANOVA followed by Tukey’s pairwise comparison post hoc test. For non-normally distributed data, Brown–Forsythe and Welch ANOVA followed by the Games-Howell multiple comparisons test was used. For the comparison of the two groups, Student’s unpaired t-test assuming a two-tailed distribution with Welch’s correction was used. Unless otherwise noted, all error bars represent s.e.m. The number of biological replicates and statistical analyses for each experiment were indicated in the figure legends. Independent experiments were performed at least in triplicates.

## Results

### *Mll4* deletion in myofiber alters CSA and slow fiber composition in males, but not in female mice

**Fig. 1:**
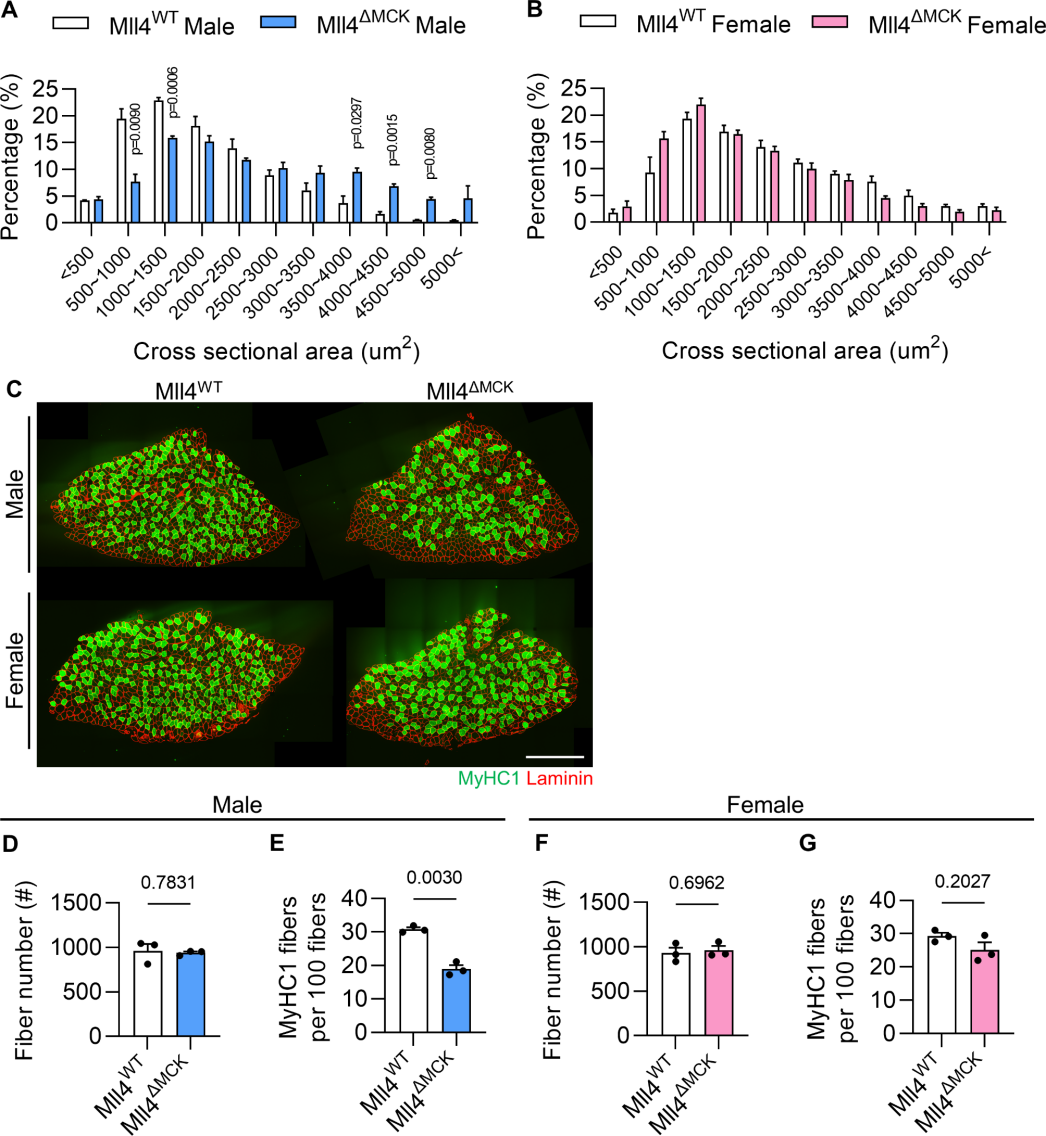
Fiber CSA and fiber type composition in adult Mll4^WT^ and Mll4^ΔMCK^ mice. (**A**-**B**) Percentage of myofibers within each indicated range of CSA in TA muscle of Mll4^ΔMCK^ and Mll4^WT^ mice. *n* = 3 mice for each genotype. (**C**) Representative image of soleus muscles immunolabeled with anti-MyHC1 (green) and anti-laminin (red). Scale bar, 500 μm. (**D**, **F**) The fiber number of whole TA muscles from Mll4^WT^ and Mll4^ΔMCK^ mice of both males and females. *n* = 3 mice for each genotype. (**E**, **G**) The percentage of slow fiber among total fiber of soleus muscles from Mll4^WT^ and Mll4^ΔMCK^ mice of both males and females. *n* = 3 mice for each genotype. Data are presented as mean ± SEM of biological replicates. Statistical analyses were performed using unpaired t-test with Welch’s correction

Previously, Liu and colleagues [23] reported that the deletion of *Mll4* in developing myofibers did not impact overall muscle mass but resulted in changes to muscle characteristics, including an increased cross-sectional area (CSA) and a shift from slow to fast fiber types. However, the results were derived exclusively from male mice. Given that Mll4 is expressed in both sexes (Supplementary Fig. 1A), we investigated whether female mice would exhibit a similar phenotype to males following *Mll4* ablation. We crossed *Mll4*^*f/f*^ mice that carry loxP sites of the *Mll4* gene [19], with *MCK*^*Cre/+*^ mice [28], which produced Mll4^ΔMCK^ mice (*MCK*^*Cre/+*^; *Mll4*^*f/f*^) [23]. By performing quantitative real-time PCR (qRT-PCR), the deletion of *Mll4* was confirmed in 8-week-old Mll4^ΔMCK^ myofibers (Supplementary Fig. 1B-C). Muscle weight remained unchanged in 8-week-old Mll4^ΔMCK^ mice, regardless of sex (Supplementary Fig. 1D-E). As reported [23], male Mll4^ΔMCK^ mice displayed increased CSA (Fig. 1A) and fiber type shifts toward a decreased ratio of slow-twitch fiber in soleus muscle (Fig. 1C-E). In contrast, no significant differences in these characteristics were observed in Mll4^ΔMCK^ female mice compared to Mll4^WT^ female mice (Fig. 1B and C, and 1F-G), suggesting that the alterations in CSA and fiber type composition following the deletion of Mll4 in myofibers may represent a phenotype selectively characteristic of male musculature, potentially attributable to male-specific factors or microenvironments.

### MuSC depletion in adult Mll4^ΔMCK^ mice

**Fig. 2:**
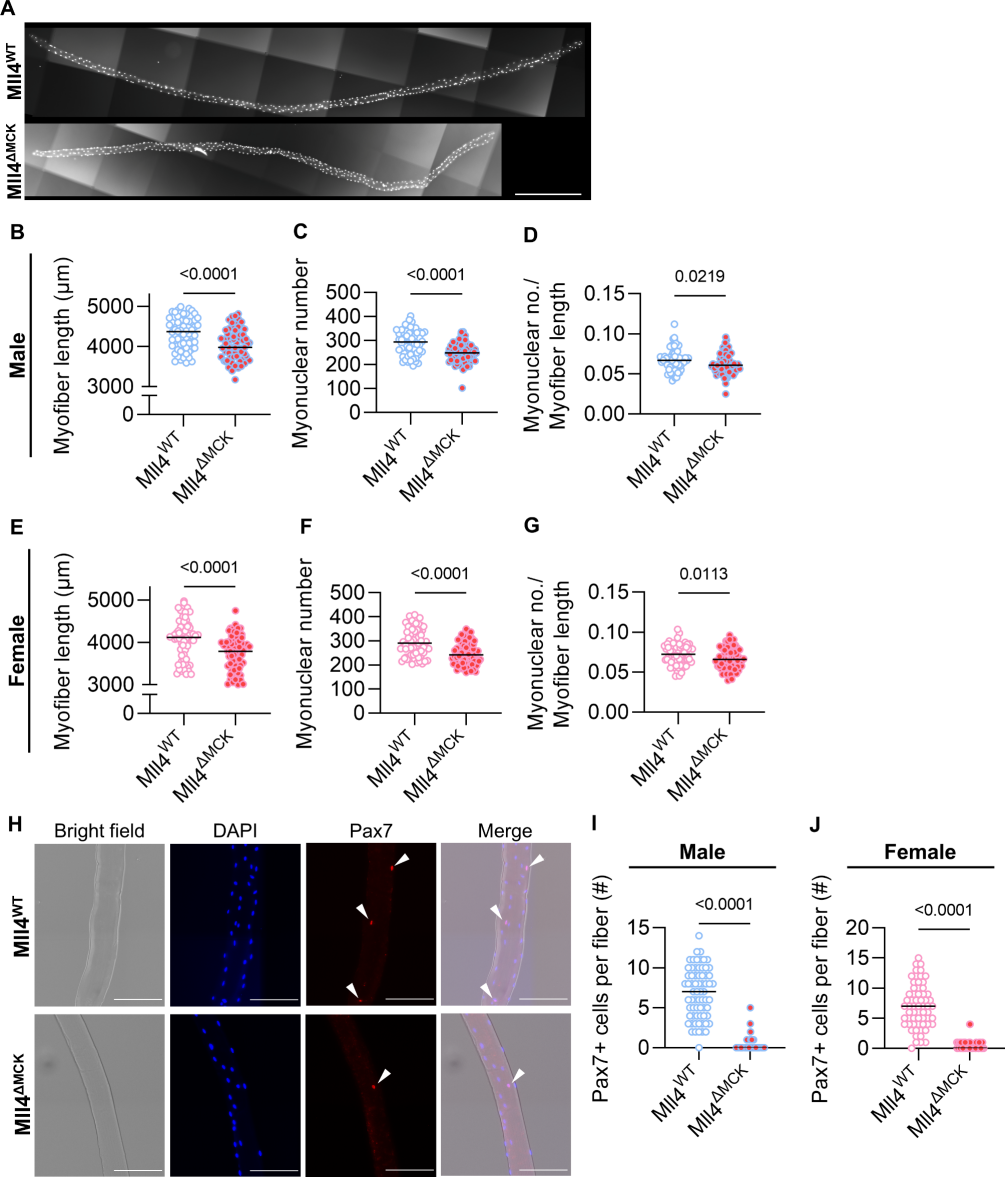
Altered myofiber phenotype of 8-week-old Mll4^ΔMCK^ mice. (**A**) Representative image of the isolated single myofiber. DAPI staining was applied to visualize nuclei. Scale bar, 500 μm. (**B**, **E**) Myofiber length, (**C**, **F**) myonuclei accretion, and (**D**, **G**) myonuclear density were quantified. (**H**) Immunocytochemistry of isolated myofibers of 4-week-old Mll4^ΔMCK^ and Mll4^WT^ mice with DAPI (blue) and anti-Pax7 (red). MuSCs are marked with arrowheads. Scale bars, 100 μm. (**I**, **J**) Pax7^+^ MuSC number per fiber of Mll4^WT^ and Mll4^ΔMCK^ mice of both genders. (**B**-**G**, and **I**-**J**) *n* = 3 mice for each genotype; >20 fibers per mouse was quantified. Data are presented as mean ± SEM of biological replicates. Statistical analyses were performed using unpaired t-test with Welch’s correction

Unexpectedly, when isolating single myofibers to prove the ablation of Mll4 in Mll4^ΔMCK^ mice (Fig. 2A), we observed that myofiber length was significantly shorter in both male and female Mll4^ΔMCK^ mice compared to those of controls (Fig. 2B and E). Since shortened myofibers may be due to a reduced number of myonuclei [29], we quantified myonuclear number, which was markedly decreased in Mll4^ΔMCK^ EDL myofibers compared to the controls (Fig. 2C and F). To assess whether myonuclear density was also reduced, we calculated the ratio of myonuclear number to myofiber length. This analysis revealed a decrease in myonuclear density in Mll4^ΔMCK^ myofibers (Fig. 2D and G).

During postnatal development in mice, myofiber length and myonuclear number increase rapidly through myoblast fusion until puberty and then become relatively stable at the adult stage when postnatal myogenesis ceases [1]. Thus, reduced myonuclear density in adult Mll4^ΔMCK^ mice might be due to impaired myoblast fusion, defective MuSC differentiation, or even MuSC depletion. To address this issue, we first assessed the number of MuSCs in *Mll4*-deleted myofibers (Fig. 2H). Intriguingly, Pax7^+^ MuSCs greatly decreased in the myofibers of both male and female 8-week-old Mll4^ΔMCK^ mice (Fig. 2I-J). Our data suggest that MuSC depletion in *Mll4*-lacking myofibers during postnatal myogenesis resulted in decreased myonuclei accretion and myofiber growth, and that *Mll4* in myofiber may play an important role in maintaining the MuSC population, irrespective of gender.

### MuSC depletion in *Mll4* deleted muscle during postnatal muscle growth

**Fig. 3:**
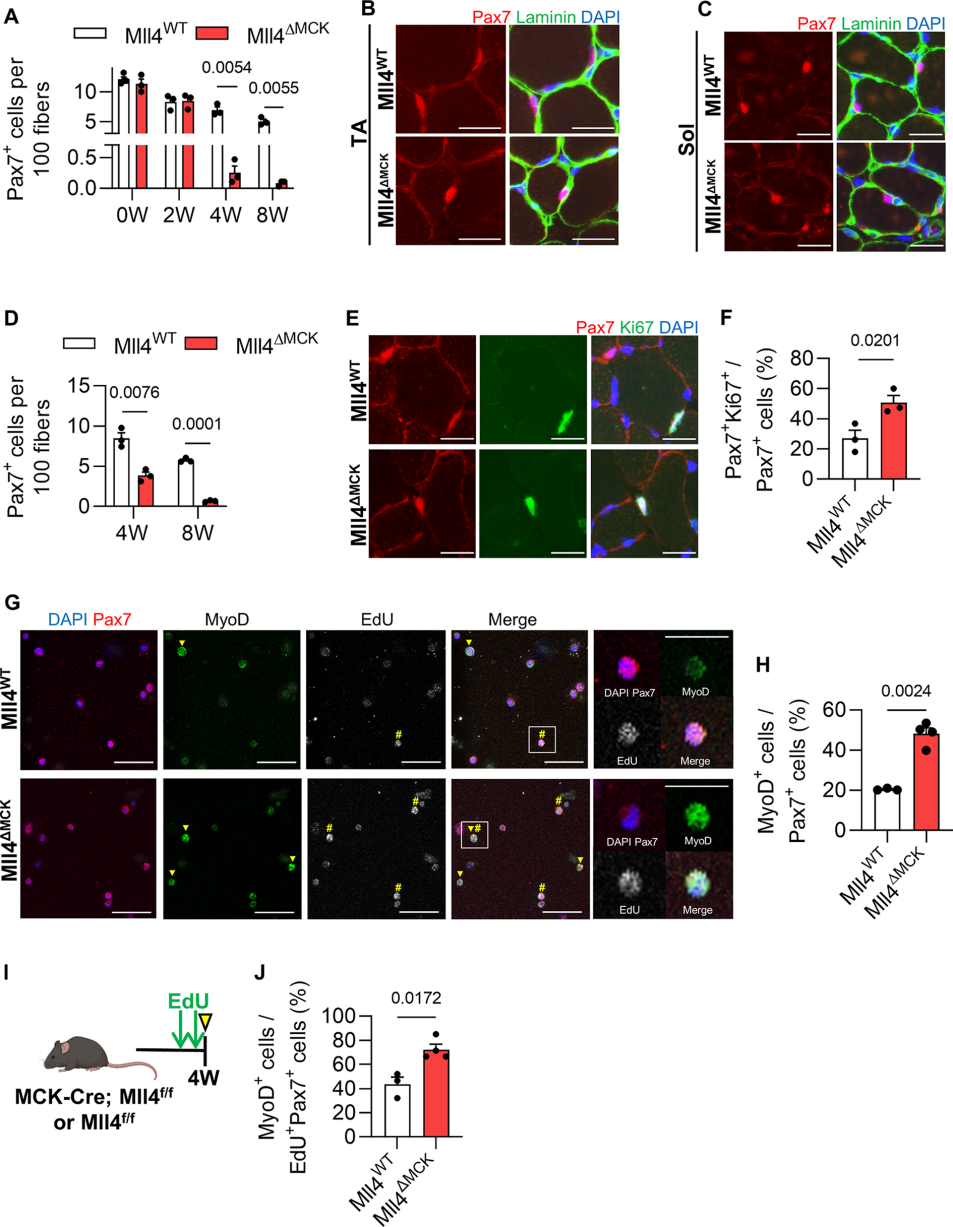
MuSC depletion due to increased population of differentiating myoblasts in 4-week-old Mll4^ΔMCK^ muscles. (**A**) Pax7^+^ MuSC number per 100 fibers was quantified in TA muscles of 0, 2, 4, and 8-week-old Mll4^ΔMCK^ and littermate control mice. Immunohistochemistry on TA (**B**) and soleus (**C**) muscle section with DAPI (blue), anti-laminin (green), and anti-Pax7 (red). Scale bars, 20 μm. (**D**) Pax7^+^ MuSC number per 100 fibers was quantified in soleus muscles of 4 and 8-week-old Mll4^ΔMCK^ and littermate control mice. (**E**) Immunohistochemistry on 4 W TA muscle section with DAPI (blue), anti-Ki67 (green), and anti-Pax7 (red). Scale bars, 20 μm. (**F**) Pax7^+^Ki67^+^ cell number per total Pax7^+^ cells of 4 W TA muscle. (**G**) Immunocytochemistry of sorted MuSCs with DAPI (blue), anti-Pax7 (red), anti-MyoD (green), and EdU (white – pseudo color for Alexa Fluor 647). MyoD^+^ cells and EdU^+^ cells are marked with arrowheads and sharps, respectively. Scale bars, 20 μm. For enlarged images, Scale bars represent 10 μm. (**H**) MyoD^+^ cell number per total Pax7^+^ cells. (**I**) Schematic diagram of EdU treatment. (**J**) MyoD^+^ cell number per EdU^+^Pax7^+^ cells. (**A**, **D**, **F**) *n* = 3–4 mice for each genotype. (**H**, **J**) *n* = 3–4 mice for each genotype; >200 sorted MuSCs per mouse were quantified. (**A**, **D**, **F**, **H**, **J**) Data are presented as mean ± SEM of biological replicates. Statistical analyses were performed using unpaired t-test with Welch’s correction

MuSCs that have actively proliferated during the juvenile stage enter a quiescent state at puberty to establish a reserve stem cell pool in adult muscles [2]. To investigate if the deletion of Mll4 in myofibers affects MuSC number during postnatal myogenesis, we conducted a histological analysis to quantify Pax7-positive cells in TA muscles during and after postnatal myogenesis. Considering that MCK-Cre mediated ablation of *Mll4* occurs after 7 days of birth [23], 0-week-old perinatal muscles were expected to have comparable MuSC numbers between Mll4^WT^ and Mll4^ΔMCK^ muscles. The number of MuSCs remained consistent between the control and Mll4^ΔMCK^ muscles until 2 weeks of age (Fig. 3A). However, a decrease of Pax7^+^ MuSCs was prominent in the pubertal 4-week-old Mll4^ΔMCK^ TA muscle, with a further decline noted in the 8-week-old muscle (Fig. 3A and B). This indicates that the deletion of myofiber-specific *Mll4* disrupts the MuSC number during postnatal myogenesis, resulting in a depletion of the population in adult muscles.

TA and EDL muscles primarily consist of fast-twitch type 2 fibers [30]. To examine if MuSC depletion occurs in slow-twitch type 1 fiber-rich muscles, the soleus muscle was analyzed. Undoubtedly, the Pax7-positive MuSC number was reduced in the pubertal 4-week-old muscles and further diminished in the adult 8-week-old soleus muscles of Mll4^ΔMCK^ mice (Fig. 3C and D).

During puberty, cycling MuSCs exit the cell cycle and contribute to quiescent MuSC populations [2]. To test if the reduced MuSC in Mll4^ΔMCK^ mice was due to impaired cell cycle exit in cycling pubertal MuSCs, we quantified Ki67-positive MuSCs in the 4-week-old Mll4^ΔMCK^ mice. Compared to the control, Mll4^ΔMCK^ mice showed a twofold increase in proliferating Ki67^+^Pax7^+^ MuSCs (Fig. 3E and F). To investigate if these proliferating MuSCs enter the differentiation state, we isolated MuSCs via cytometry (Supplementary Fig. 2A-B) and quantified MyoD-positive MuSCs. To clarify, cells that show low Pax7 expression coupled with high MyoD expression were classified as ‘MyoD-positive’ cells. Compared to the Mll4^WT^ mice, Mll4^ΔMCK^ mice showed an increase in MyoD-positive MuSCs (Fig. 3G-H). Furthermore, to label cycling MuSCs, EdU was treated for 2 consecutive days before isolating MuSCs (Fig. 3I). Notably, the number of MyoD-positive cells also increased among cycling MuSCs (EdU^+^Pax7^+^) in Mll4^ΔMCK^ mice (Fig. 3J). This indicates that while normal MuSCs exit the cell cycle and enter a quiescent state during puberty, MuSCs in Mll4^ΔMCK^ mice maintain their cell cycle and differentiate into committed myoblasts. Altogether, the deletion of Mll4 in myofibers leads to the loss of MuSCs during postnatal muscle growth.

### Deletion of Mll4 in adult myofibers does not alter muscle CSA and fiber type composition

**Fig. 4:**
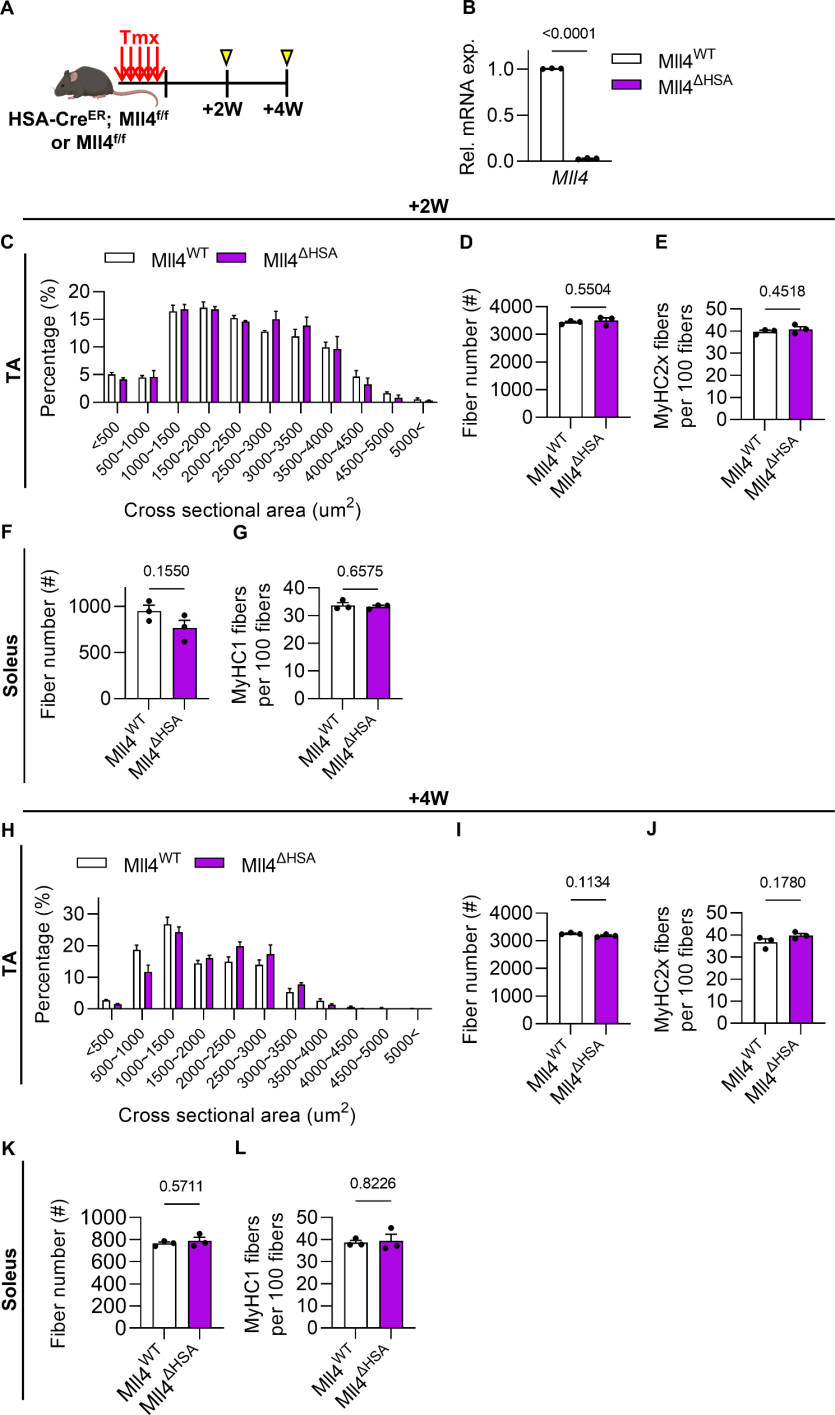
Induced deletion of Mll4 in the adult stage does not impact myofiber maintenance. (**A**) Schematic diagram of mouse preparation. (**B**) qRT-PCR analysis of myofibers to confirm the downregulation of the *Mll4* gene in + 2 W Mll4^ΔHSA^ mice. (**C**) Percentage of myofibers within each indicated range of CSA, (**D**) gross fiber number, and (**E**) percentage of MyHC2x fibers in TA muscle of Mll4^WT^ and + 2 W Mll4^ΔHSA^ mice. (**F**) Gross fiber number and (**G**) percentage of MyHC1 fibers in soleus muscle of Mll4^WT^ and Mll4^ΔHSA^ mice. (**H**) Percentage of myofibers within each indicated range of CSA, (**I**) gross fiber number, and (**J**) percentage of MyHC2x fibers in TA muscle of Mll4^WT^ and + 4 W Mll4^ΔHSA^ mice. (**K**) Gross fiber number (**L**) and percentage of MyHC1 fibers in soleus muscle of Mll4^WT^ and + 4 W Mll4^ΔHSA^ mice. (**B**-**L**) *n* = 3 mice for each genotype. Data are presented as mean ± SEM of biological replicates. Statistical analyses were performed using unpaired t-test with Welch’s correction

Following postnatal myogenesis, the adult muscle tissue reaches a steady state characterized by the cessation of myofiber growth and the entry of MuSCs into a quiescent phase. To investigate if induced ablation of *Mll4* in muscles after postnatal myogenesis would affect myofiber maintenance, we examined muscle features such as CSA distribution and fiber type composition of *Mll4* ablated adult muscles. Adult *HSA*^*MerCreMer/+*^; *Mll4*^*f/f*^ mice (Mll4^ΔHSA^) were treated with tamoxifen for consecutive 5 days to induce deletion of the *Mll4* gene in myofibers (Fig. 4A). This resulted in the ablation of the *Mll4* gene in the myofibers of Mll4^ΔHSA^ mice after 2 weeks of tamoxifen administration (Fig. 4B). The histological analysis showed that CSA distribution, fiber number, and fast-twitch (MyHC2x) fiber composition in the TA muscles of Mll4^ΔHSA^ mice did not change following 2 weeks (Fig. 4C-E, Supplementary Fig. 3A) and even 4 weeks (Fig. 4H-J, Supplementary Fig. 3B) of *Mll4* ablation, compared to the control mice. Similarly, in the soleus muscle, the composition of slow-twitch (MyHC1) fibers also remained unchanged after both 2 weeks (Fig. 4F-G, Supplementary Fig. 3A) and 4 weeks (Fig. 4K-L, Supplementary Fig. 3B) of *Mll4* ablation in Mll4^ΔHSA^ mice. This indicates that the induced deletion of *Mll4* in adult muscles does not affect myofiber maintenance and intracellular features such as fiber CSA and fiber type composition regardless of gender.

### Mll4 deletion in myofibers does not affect exercise capacity

Since MLL4 functions as an enhancer activator [19], its deletion may disrupt various gene transcription processes. Although the deletion of Mll4 in adult muscles does not impact myofiber characteristics such as CSA and fiber type composition, we investigated whether the deletion affects exercise capacity. To test this, tamoxifen-treated mice were accustomed to chronic exercise training for 5 weeks (hereafter, Mll4^WT − EX^ and Mll4^ΔHSA−EX^) (Supplementary Fig. 4A). The protocol of the chronic exercise provides prolonged contractions of muscles, promoting adaptations such as increased muscle mass while minimizing exercise-induced muscle damage [31]. In TA muscles, CSA distribution and fast fiber composition remained consistent between Mll4^WT − EX^ and Mll4^ΔHSA−EX^ mice. In addition, slow fiber composition was unchanged in Mll4^ΔHSA−EX^ soleus muscle (Supplementary Fig. 4B-G).

To assess whether exercise capacity was affected by Mll4 deletion, we measured grip strength and endurance running capability. In line with the observed similarity in CSA and fiber type composition, Mll4^ΔHSA−EX^ mice showed comparable grip strength (Supplementary Fig. 4H) and endurance running capability (Supplementary Fig. 4I-J) relative to the control group. To investigate whether the expression of genes related to fiber type and metabolism was altered in Mll4^ΔHSA−EX^ mice, we conducted qRT-PCR analyses. Transcriptional profiling revealed that before exercise training, the genes were generally downregulated in Mll4^ΔHSA^ mice (Supplementary Fig. 5A-B). Notably, no particular gene exhibited higher expression levels that could induce a shift in fiber type composition or metabolic activity. After 5 weeks of chronic exercise, the expression of genes related to fiber type and metabolism of Mll4^ΔHSA−EX^ mice also showed overall downregulation, with the exception that certain slow-twitch muscle genes were marginally upregulated (Supplementary Fig. 5C). Collectively, the results suggest that the induced knockout of *Mll4* in myofibers at the adult stage does not influence exercise capacity or muscle characteristics, such as CSA and fiber type composition, even after physiological exercise stimulation.

### Loss of adult MuSCs in Mll4^ΔHSA^ mice

**Fig. 5:**
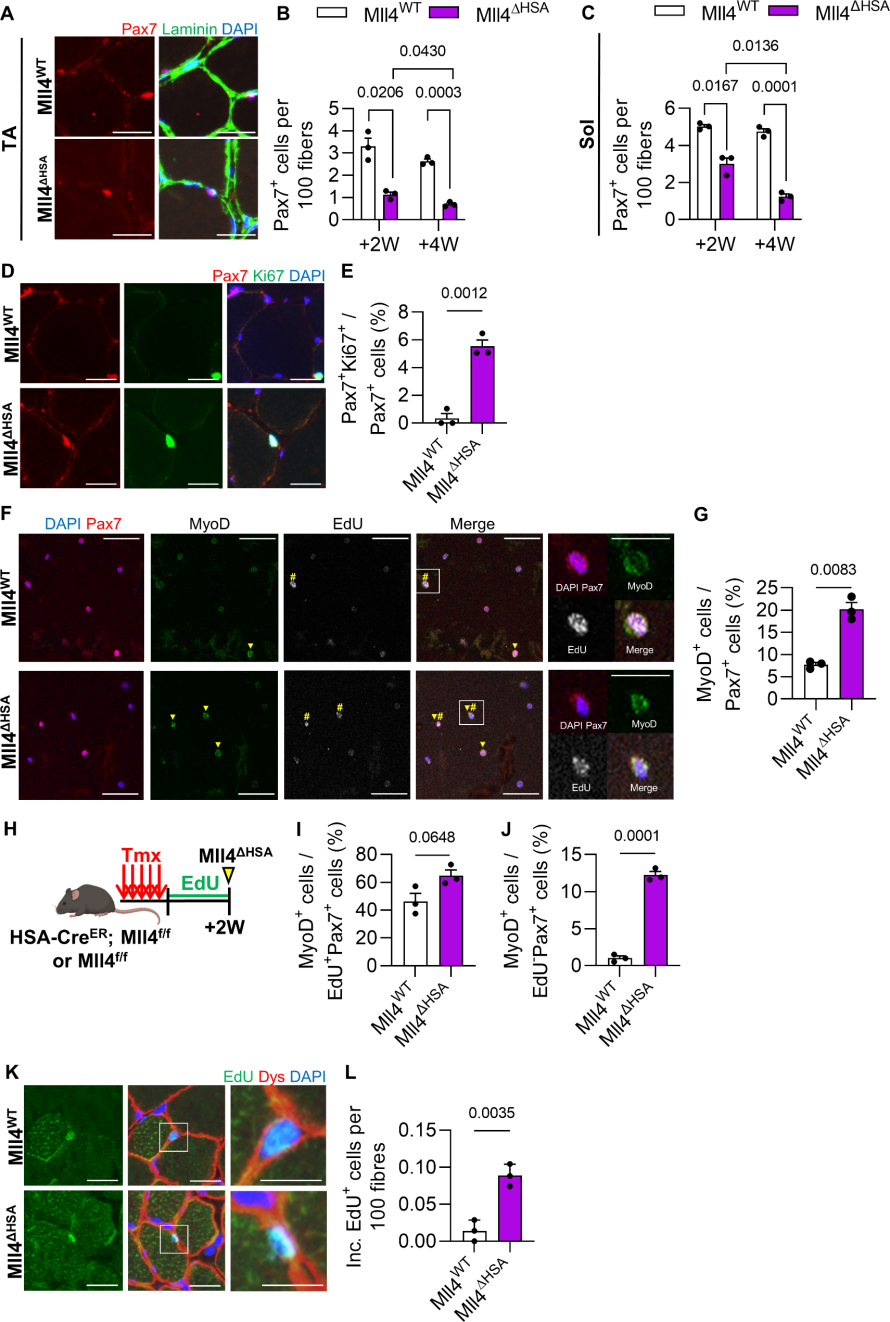
Severe MuSC deprivation in *Mll4* deleted adult myofibers. (**A**) Immunohistochemistry on TA muscle section with DAPI (blue), anti-laminin (green), and anti-Pax7 (red). (**B**) Pax^+^ MuSC number per 100 fibers of Mll4^WT^ and Mll4^ΔHSA^ TA and (**C**) soleus muscle. Scale bars, 20 μm. (**D**) Immunohistochemistry on TA muscle section with DAPI (blue), anti-Ki67 (green), and anti-Pax7 (red). Scale bars, 20 μm. (**E**) Pax7^+^Ki67^+^ cell number per total Pax7^+^ cells. (**F**) Immunocytochemistry of sorted MuSCs with DAPI (blue), anti-Pax7 (red), anti-MyoD (green), and EdU (white – pseudo color for Alexa Fluor 647). MyoD^+^ cells and EdU^+^ cells are marked with arrowheads and sharps, respectively. Scale bars, 20 μm. For enlarged images, Scale bars represent 10 μm. (**G**) MyoD^+^ cell number per total Pax7^+^ cells. (**H**) Schematic diagram of EdU treatment. (**I**) MyoD^+^ cell number per EdU^+^Pax7^+^ cells, and (J) MyoD^+^ cell number per EdU^−^Pax7^+^ cells were quantified. (**K**) Immunohistochemistry on TA muscle section with DAPI (blue), EdU (green), and anti-dystrophin (red). Scale bars, 20 μm. For enlarged images, Scale bars represent 10 μm. (**L**) The number of fiber incorporated EdU^+^ cells per 100 fibers. (**B**, **C**, **E**, and **L**) *n* = 3–4 mice for each genotype. (**G**, **H**-**I**) *n* = 3–4 mice for each genotype; >200 sorted MuSCs per mouse were quantified. (**B**, **C**, **E**, **L**, **G**, **H**-**I**) Data are presented as mean ± SEM of biological replicates. Statistical analyses were performed using unpaired t-test with Welch’s correction

To investigate whether the deletion of *Mll4* in adult myofibers disturbs the quiescence of MuSCs, TA and soleus muscles were analyzed to quantify Pax7-positive MuSCs. Mll4^ΔHSA^ mice showed depletion of MuSCs in both muscles (Fig. 5A-C). To assess whether the loss of adult quiescent MuSCs in Mll4^ΔHSA^ mice is associated with their entry into the cell cycle, we quantified Ki67-positive MuSCs in the TA muscles. In Mll4^WT^ TA muscles, there was a negligible presence of Ki67^+^Pax7^+^ cells, whereas Mll4^ΔHSA^ muscles showed an increase of Ki67^+^Pax7^+^ cells (Fig. 5D-E). This suggests that ablation of *Mll4* in myofibers at the adult stage causes MuSCs to exit quiescence and enter the cell cycle, leading to the depletion of MuSCs. To examine if adult Mll4^ΔHSA^ muscle shows an increased number of differentiated MuSCs, we performed an immunocytochemistry assay on sorted MuSCs (Supplementary Fig. 2C-D) to quantify MyoD-positive cells. Compared to the Mll4^WT^ MuSCs, Mll4^ΔHSA^ MuSCs showed an increased number of MyoD-expressing cells (Fig. 5F-G). For detecting MuSCs that entered the cell cycle, we treated EdU for 2 weeks in Mll4^ΔHSA^ mice (Fig. 5H). This long-term EdU labeling method was applied to identify the scarcely dividing MuSCs in adult muscle tissue [32]. We found that the population of MyoD-expressing cells among EdU-positive, dividing MuSCs was also increased in Mll4^ΔHSA^ muscles (Fig. 5I). On the other hand, given that adult quiescent MuSCs can differentiate without entering the cell cycle [32], we also quantified MyoD-expressing cells among EdU-negative, non-dividing MuSCs. Interestingly, Mll4^ΔHSA^ MuSCs showed an increased number of MyoD-positive cells among EdU-negative MuSCs (Fig. 5J). This indicates that the deletion of Mll4 in adult muscles causes adult MuSCs to lose their quiescence and undergo differentiation, either with or without dividing.

To test whether the differentiated myogenic progeny of Mll4^ΔHSA^ muscles fuse into myofibers, we conducted a histological analysis and quantified EdU-positive nuclei located on the inner side of dystrophin structure [32] (Fig. 5K). While the number of fiber-incorporating EdU-positive nuclei was extremely low in control muscles, it was sevenfold higher in Mll4^ΔHSA^ muscles compared to the control group (Fig. 5L). This suggests that *Mll4* deficiency in adult muscles causes MuSCs to exit quiescence and undergo differentiation, with at least some, or perhaps all, of these differentiated cells subsequently fusing into myofibers. This eventually results in severe MuSC loss in adult muscles.

### Lack of *Mll4* impairs muscle regeneration following injury

**Fig. 6:**
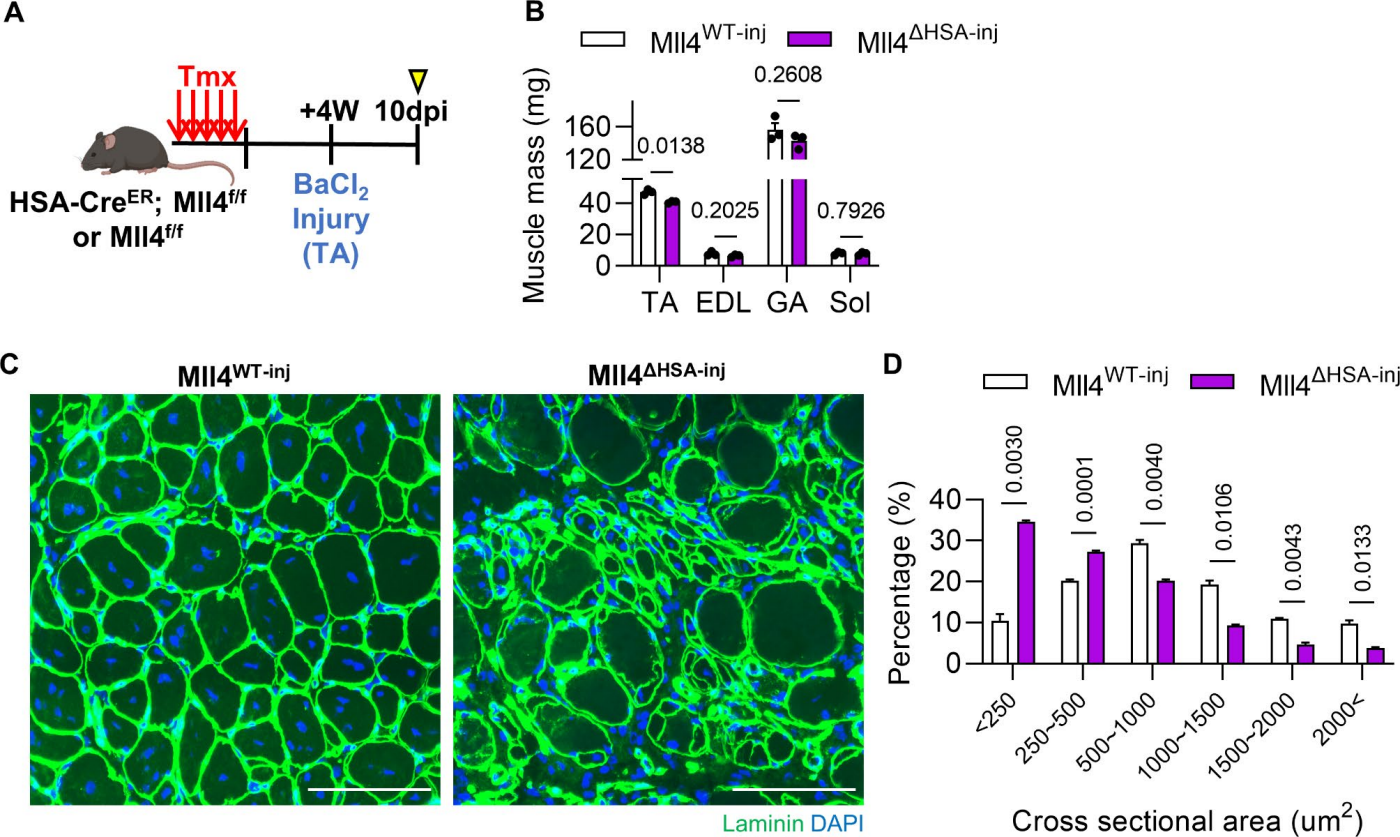
Defective muscle regeneration capacity after injury in *Mll4* deleted TA muscles. (**A**) Schematic diagram of muscle injury and mouse preparation. (**B**) Muscle mass of TA, EDL, GA, and soleus muscles of Mll4^WT − inj^ and Mll4^ΔHSA−inj^ mice. (**C**) Representative image of TA muscle labeled with anti-laminin (green) and Hoechst 33,342. Scale bars, 100 μm. (**D**) Percentage of myofibers within each indicated range of CSA in TA muscle of Mll4^WT − inj^ and Mll4^ΔHSA−inj^ mice. (**B** and **D**) *n* = 3 mice for each genotype. Data are presented as mean ± SEM of biological replicates. Statistical analyses were performed using unpaired t-test with Welch’s correction

MuSCs are the primary cell type that contributes to muscle regeneration capacity. To investigate if MuSC depletion in Mll4^ΔHSA^ mice leads to hindered muscle regeneration, we subjected TA muscles of Mll4^WT^ and Mll4^ΔHSA^ mice to injury using BaCl_2_ (hereafter, Mll4^WT − inj^ and Mll4^ΔHSA−inj^) (Fig. 6A). Muscles were analyzed 10 days post-injury, as the majority of regenerating fibers are restored [33]. TA muscles of Mll4^ΔHSA−inj^ mice showed reduced muscle mass compared to that of Mll4^WT − inj^ mice, while adjacent muscles such as the EDL, GA, and soleus remained unaffected (Fig. 6B). Histological analysis of muscle sections revealed active muscle regeneration in Mll4^WT − inj^ muscles, as indicated by the predominance of myofibers with centrally located nuclei and relatively homogenous fiber sizes. Conversely, Mll4^ΔHSA−inj^ muscle was observed with disorganized tissue architecture with residual damaged fibers that failed to undergo effective regeneration. (Fig. 6C). Moreover, Mll4^ΔHSA−inj^ mice showed reduced CSA of regenerating fibers (Fig. 6D). These results suggest that lack of MuSCs due to the deletion of myofiber-specific Mll4 resulted in severely impinged muscle regeneration capacity.

### Mll4 in myofibers affects the MuSC niche by regulating Notch ligand expression

**Fig. 7:**
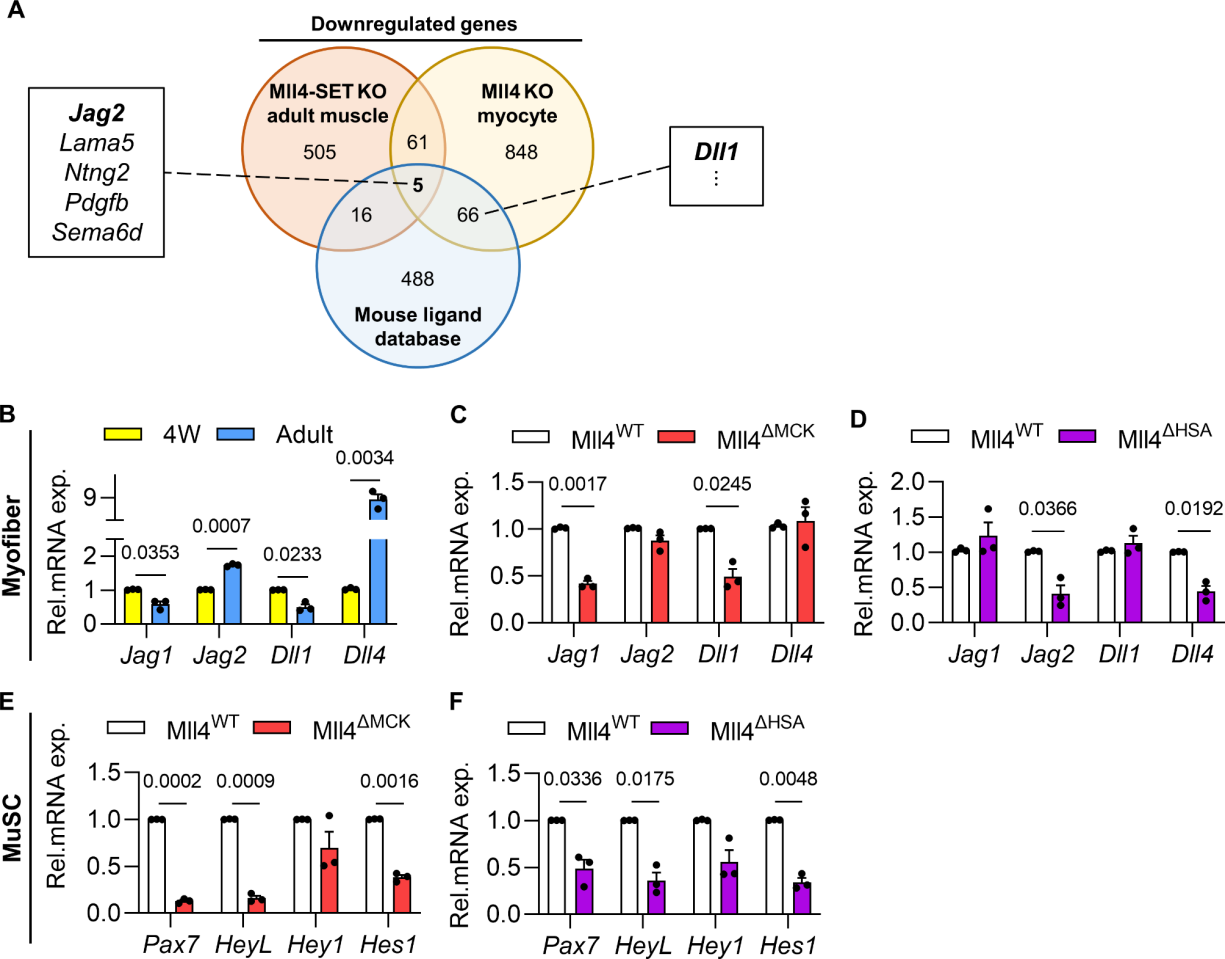
Altered Notch ligand expression in *Mll4* deleted myofibers, leading to downregulated notch signaling in MuSCs. (**A**) Evaluation of three public databases proved downregulation of Notch ligands in *Mll4*-deleted muscle and myocyte. (**B**) qRT-PCR analysis to compare Notch ligand expression in pubertal (4 W) and adult myofibers. (**C**-**D**) qRT-PCR analysis to quantify mRNA expression of Notch ligands in myofibers of 4-week-old Mll4^ΔMCK^ mice and adult Mll4^ΔHSA^ mice. (**E**-**F**) qRT-PCR of canonical Notch effectors confirmed general downregulation of Notch signaling in Mll4^ΔMCK^ and Mll4^ΔHSA^ MuSCs. (**B**-**F**) *n* = 3 mice for each genotype. Data are presented as mean ± SEM of biological replicates. Statistical analyses were performed using unpaired t-test with Welch’s correction

To explore how *Mll4* in myofibers may have affected the MuSC niche, we screened for downstream effector candidate genes by analyzing public datasets. For one, we analyzed data curated by Liu et al. [23]. This provided a list of downregulated genes in muscle from MLL4-SET-knockout (KO) mice, where the enzymatic SET domain of MLL4 is ablated, compared to control mice. In addition, we analyzed data from Lee et al. [19], to obtain the list of downregulated genes in cultured, differentiated Mll4-KO myocytes versus control. Sixty-six genes were identified as commonly downregulated from the two datasets. Since myofiber can directly regulate the MuSC niche via signaling through ligand-receptor interactions [34], we then identified ligands from the 66 candidate genes by comparing them to the mouse ligand database. Consequently, 5 genes were identified as ligand-coding genes that are downregulated by Mll4 KO in both whole muscle and differentiated myocytes. To our surprise, the Notch ligand Jag2 was among the 5 candidate genes. Also, Dll1, another Notch ligand, was identified as downregulated in Mll4 KO myocytes. (Fig. 7A).

For MuSCs, Notch signaling is a major signaling pathway that maintains the stem cell pool. When the Notch downstream effector Rbpj is deleted in adult MuSCs, which are predominantly in a quiescent state, they exit the quiescent state and undergo aberrant differentiation [32, 35]. Myofiber-specific deletion of Dll4, a Notch ligand that is mainly expressed in adult myofibers, induces premature differentiation of MuSCs, resulting in a reduced number of stem cells [17, 36]. These studies suggest that the maintenance of MuSC quiescence is highly dependent on Notch signaling between MuSCs and myofibers. Considering that the depletion of MuSCs was observed in both Notch signaling-reduced MuSCs and myofiber-specific *Mll4-*deleted (Mll4^ΔMCK^ and Mll4^ΔHSA^) MuSCs, we sought to validate the downregulation of Notch ligands in Mll4^ΔMCK^ and Mll4^ΔHSA^ mice.

A previous study found that *Dll4* is highly expressed in adult myofibers [17]. In addition, we reported that myofibers of 4-week-old mice exhibited robust expression of *Dll1* and *Jag1* proteins [2]. Considering that Notch ligands have a fluctuating expression pattern in muscles throughout the developmental stages, we compared mRNA quantity for Notch ligands in the myofibers of wild-type pubertal 4-week-old and adult mice (Fig. 7B). Interestingly, genes having dominant expression during each time point correlated with genes that were downregulated due to *Mll4* depletion in muscle fibers. While expression of major Notch ligands of pubertal 4-week-old myofibers – *Jag1* and *Dll1* – decreased in 4-week-old Mll4^ΔMCK^ myofibers (Fig. 7C), the primary Notch ligands of adult myofibers – *Jag2* and *Dll4* – decreased in adult Mll4^ΔHSA^ myofibers (Fig. 7D). In other words, *Mll4* insufficiency in myofibers disturbed Notch ligand expression that is dominant in each pubertal or adult muscle.

To test if Notch signaling is indeed reduced in 4-week-old Mll4^ΔMCK^ and adult Mll4^ΔHSA^ MuSCs, the mRNA levels of canonical Notch effectors – *HeyL*,* Hey1*, and *Hes1* – were quantified via qRT-PCR. As expected, the overall expression of genes mentioned above was downregulated in both Mll4^ΔMCK^ and adult Mll4^ΔHSA^ MuSCs (Fig. 7E-F).

Considering the molecular feature of MLL4, we analyzed public ChIP-seq data of MLL4 in myocytes [19], to examine whether it may modulate the transcription of Notch ligands on the chromosomal level. This revealed the genomic binding of MLL4 on *Dll1* and *Jag2* gene loci, where *Mll4* deletion reduced H3K4me1 and H3K27ac levels on enhancers for both *Dll1* and *Jag2* genes (Supplementary Fig. 6A-B). This implicates the possibility of MLL4 directly regulating the induction of different Notch ligand gene expressions. Taken together, MLL4 can control diverse Notch ligand expression in myofibers, which is necessary for regulating the MuSC niche during and after postnatal myogenesis.

## Discussion

Skeletal muscle has a resilient characteristic due to its resident stem cell populations. Thus, uncovering the mechanism of regulating MuSC fate is crucial for understanding the biological process of developmental and regenerative myogenesis. In this paper, we explored the role of *Mll4* in myofibers regarding the MuSC state regulation and discovered that myofiber-expressed *Mll4* is important for maintaining MuSCs in both muscles during and after postnatal myogenesis. In the pubertal Mll4^ΔMCK^ muscle, lack of *Mll4* in myofibers resulted in increased population of differentiating myogenic cells, leading to a decrease of MuSCs. Furthermore, induced ablation of *Mll4* in adult myofibers resulted in the quiescence exit of MuSCs, which also caused dramatic depletion of MuSCs. During postnatal myogenesis, juvenile MuSCs constantly proliferate for muscle development [1, 2]. This proliferating cell population decreases due to cell cycle exit during puberty to establish a reserve pool of quiescent MuSCs in adult muscles [3, 4]. Our findings indicate that Mll4 in myofibers are critical for maintaining MuSCs in pubertal muscles, where cycling MuSCs begin to enter quiescence, as well as in adult muscles, where MuSCs remain in a quiescent state. This suggests that *Mll4* plays a critical role in myofibers by creating a microenvironment that supports the maintenance of a healthy population of MuSCs in muscle tissue.

The ability to maintain an adequate number of MuSCs is crucial regardless of gender and age. In this study, we elucidate two critical properties of *Mll4* in preserving the stemness of MuSCs. First, *Mll4* regulates the MuSC number in both sexes. Skeletal muscle exhibits sexual dimorphism in terms of mass, fiber type composition, and contractility attributed to variations in gene expression and hormonal profiles between genders [37, 38]. These differences may have contributed to the disparate muscle phenotypes after the deletion of myofiber-*Mll4* in male and female mice (Fig. 1C-I). However, gender did not influence the extent of MuSC depletion resulting from *Mll4* ablation. Secondly, Mll4 regulates MuSC quiescence across different developmental stages, including both during and after postnatal myogenesis. Previous research indicated that myofiber-specific deletion of *Mll4* led to a slow-to-fast fiber type shift [23]. However, our findings reveal that this phenotype is not present following *Mll4* deletion in adult muscle tissue. The expression of genes related to both slow and fast muscle fibers demonstrated a broad reduction following the deletion of *Mll4*. However, since the altered expression profile was not biased toward a specific fiber type, it did not result in significant physiological changes. Therefore, we suggest that the myofiber phenotype is unlikely to undergo substantial alterations even after prolonged periods post-*Mll4* deletion. This indicates that Mll4 may play a role in the development of myofibers, but not in their maintenance during adulthood, at which developmental myogenesis is complete. Indeed, it is reported that during developmental myogenesis, Foxo/Notch signaling regulates fiber type specification, leading to a reduction in slow fibers and an increase in fast fibers when disrupted in muscle [39]. Considering that (1) this phenotype is in line with that of *Mll4*-mKO mice, as reported previously, and (2) Mll4 has the possibility of regulating the gene expression of Notch ligands, the deletion of *Mll4* might have disrupted Notch signaling in developing myofibers, leading to aberrant fiber type specification. Altogether, our data provide new insights into the role of Mll4 as a crucial regulator of the MuSC quiescence, highlighting its significance across developmental stages and irrespective of gender.

Chromatin modification of enhancers within myofibers can modulate the expression of extracellular matrix (ECM) components or growth factors, thereby indirectly influencing the MuSC niche [40, 41]. Our study suggests that MLL4, an enhancer activator, regulates Notch ligand expression in myofibers to directly control MuSC quiescence. By analyzing and validating transcriptome databases from previous studies, we verified that the expression of Notch ligands was downregulated in Mll4-KO myocyte. Moreover, an analysis of ChIP-seq data revealed genomic binding of MLL4 on Notch ligand gene loci. The downregulation of Notch ligands in myofibers led to a reduced expression of canonical Notch target genes in MuSCs. This indicates that MLL4 can regulate the signaling pathway that affects adjacent cells. This finding is particularly intriguing given that MLL4 has primarily been studied as a critical factor for activating intracellular signaling pathways, including those related to cancer and cell fate determination [19, 20, 42–44]. Specifically, in myofibers, Mll4 was reported to activate the transcription of slow-twitch genes [23]. By inspecting the physiological impact of *Mll4* deletion in myofibers on MuSCs, we revealed that *Mll4* regulates not only intracellular signaling pathways, as previously reported, but also signaling pathways that affect adjacent cells, by controlling expressions of ligand genes. This underscores the importance of exploring the potential gene-regulating activity of MLL4, which may impact other cellular processes, such as differentiation and tumorigenesis, in neighboring cells.

It has been well-established that Notch signaling is a fundamental pathway regulating the MuSC niche in prenatal and postnatal muscles to maintain an appropriate stem cell pool [11, 12]. In this study, we observed that the phenotype of the myofiber-specific Mll4-cKO was very similar to that of MuSCs with disrupted Notch signaling. MuSCs in both myofiber-specific Mll4-cKO mice and MuSC-specific Notch-cKO mice exhibit reduced expression of canonical Notch target genes [35, 45], accompanied by increased expression of MyoD and Ki67 [35, 45–47]. These changes led to a reduction in MuSC numbers during postnatal muscle development [46] and increased fusion of myoblasts with myofibers in adult muscles, which also resulted in a remarkable loss of MuSCs [32]. These findings support the hypothesis that myofiber-expressed Mll4 may regulate the expression of Notch ligands, thereby modulating Notch signaling in MuSCs and influencing their cell fate.

Previous studies reported that the myofiber is an important source of Notch ligands, sending signals to MuSCs to control their niche and hence their cell fate [2, 13, 45]. Notch ligands have distinct expression patterns in myofibers during development, affecting the MuSC niche in different ways. In this study, we investigated the Notch ligands with prevailing expression in different stages; *Jag1* and *Dll1* in pubertal myofibers, and *Jag2* and *Dll4* in adult muscle fibers. Interestingly, *Mll4* deletion in pubertal and adult myofibers disturbed the expression of Notch ligands that were principally expressed in each stage. Previously, Eliazer and colleagues reported that myofiber-specific deletion of Dll4 resulted in a reduction of MuSCs. However, the decrease in MuSCs was more pronounced when the pan-Notch regulator Mib1 was deleted from myofibers [17]. This implies the presence of a complementary Notch ligand acting as a signaling factor to maintain MuSC quiescence in adult muscles, together with Dll4. Our findings suggest that along with the well-known factor Dll4, Jag2 may be another Notch ligand in adult myofibers contributing to maintaining the Pax7^+^ quiescent stem cells, both of which were found to be regulated by Mll4. Taken together, this study suggests that MLL4 functions as a regulator that modulates the expression of various Notch ligands in myofibers during both pubertal and adult stages. This regulation is essential for the precise control of MuSC quiescence throughout developmental stages.

*Mll4* deletion notably hindered H3K4me1 and H3K27ac levels on enhancers for both the *Dll1* gene in pubertal fibers and the *Jag2* gene in adult fibers, indicating different gene regulation of MLL4 in the two developmental stages. This may be attributed to the distinct pioneer transcription factors that recruit MLL4 to induce Notch ligand expression at different developmental stages. Several transcription factors – such as CCAAT/enhancer-binding protein family, myocyte enhancer factor 2 family, and Nrf1 – are identified to bind the DNA to recruit the MLL4 complex [19, 21, 23, 48]. Depending on the cell type and differentiation stage, different transcription factors recruit MLL4 to regulate the expression of various genes. Transcription factors associated with Notch signaling have also been identified. A study on chicken embryos found that a transcription coregulator, Yap, binds to the enhancer of *Jag2* [49]. In mice, Notch1 ICD can act as a transcription activator in muscle fibers to activate the gene expression of *Jag2* and *Dll4* [50]. Following these studies, it is plausible that Yap and Notch ICD may recruit MLL4 to regulate the expression of different Notch ligands in myofibers. Further investigation is required to elucidate the molecular mechanism by which MLL4 regulates Notch ligand gene expression in myofibers. This includes identifying the specific pioneer transcription factors that interact with MLL4 across different developmental stages.

## Conclusions

Our results suggest a unique function of Mll4 in myofibers controlling MuSC state, possibly by orchestrating different Notch ligand expressions in various developmental stages. Moreover, despite skeletal muscle being known to exhibit sexual dimorphism, the role of Mll4 regulating MuSCs was valid in both male and female mice. By elucidating an additional mechanism governing MuSC maintenance, this research opens new avenues for the biological manipulation of muscle stem cells.

## Electronic supplementary material

Below is the link to the electronic supplementary material.

Supplementary Material 1: Additional file 1 (PDF)

Supplementary Material 2: Supplementary Table 1: Mouse primers used for genotyping and qRT-PCR

## Data Availability

No datasets were generated or analysed during the current study.

## Abbreviations

MuSCs: Muscle Stem Cells
Mll4: Myeloid/lymphoid or Mixed-Lineage Leukemia 4
Jag1, Jag2: Jagged 1, Jagged 2
Dll1, Dll2: Delta-like protein 1, Delta-like protein 2
TA: Tibialis Anterior
Sol: Soleus
GA: Gastrocnemius
EDL: Extensor Digitorum Longus
CSA: Cross-Sectional Area
MyHC1: Myosin Heavy Chain 1
MyHC2x: Myosin Heavy Chain 2x
