# Supplementary material for: Mll4 in skeletal muscle fibers maintains muscle stem cells
Source: Skelet Muscle. 2024 Dec 23;14:35. doi: 10.1186/s13395-024-00369-9 (PMC11665228; doi:10.1186/s13395-024-00369-9)
Supplement: Supplementary file 1 — Supplementary Material 1: Additional file 1 (PDF) [file 13395_2024_369_MOESM1_ESM.docx]

**
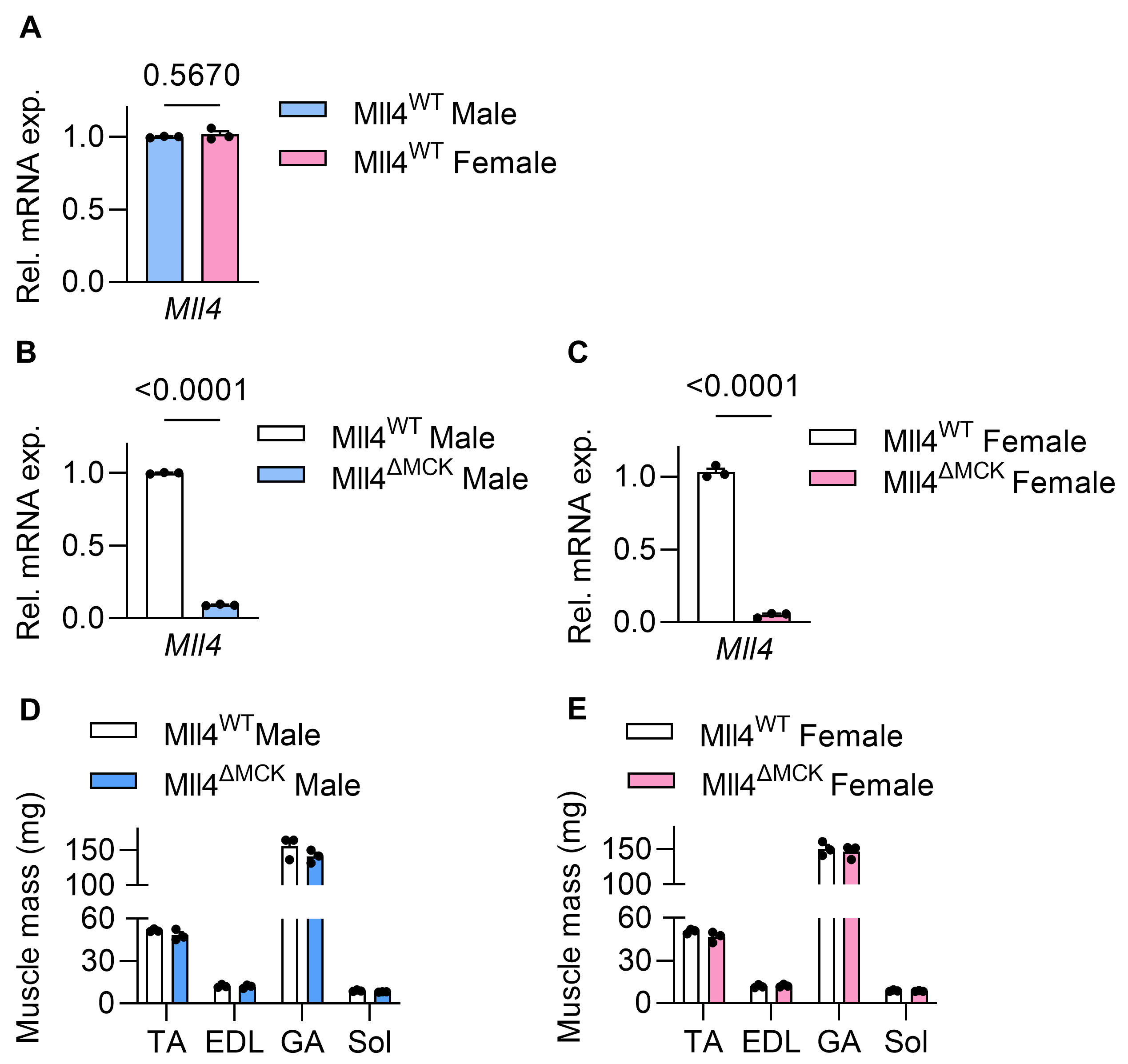
**

**Supplementary figure 1. Mll4 expression and muscle mass of Mll4^WT^ and Mll4^ΔMCK^ mice.**

**(A)** qRT-PCR analysis to detect *Mll4* expression in 8-week-old Mll4^WT^ Male and female. **(B, C)** qRT-PCR analysis to detect *Mll4* exon deletion in myofibers of male (B) and female (C) 8-week-old Mll4^ΔMCK^ and Mll4^WT^ mice. n=3 mice for each genotype. **(D, E)** Muscle mass of TA, EDL, GA, and soleus muscle from Mll4^WT^ and Mll4^ΔMCK^ mice of both males and females. n=3 mice for each genotype. Data are presented as mean ± SEM of biological replicates. Statistical analyses were performed using unpaired t-test with Welch’s correction.


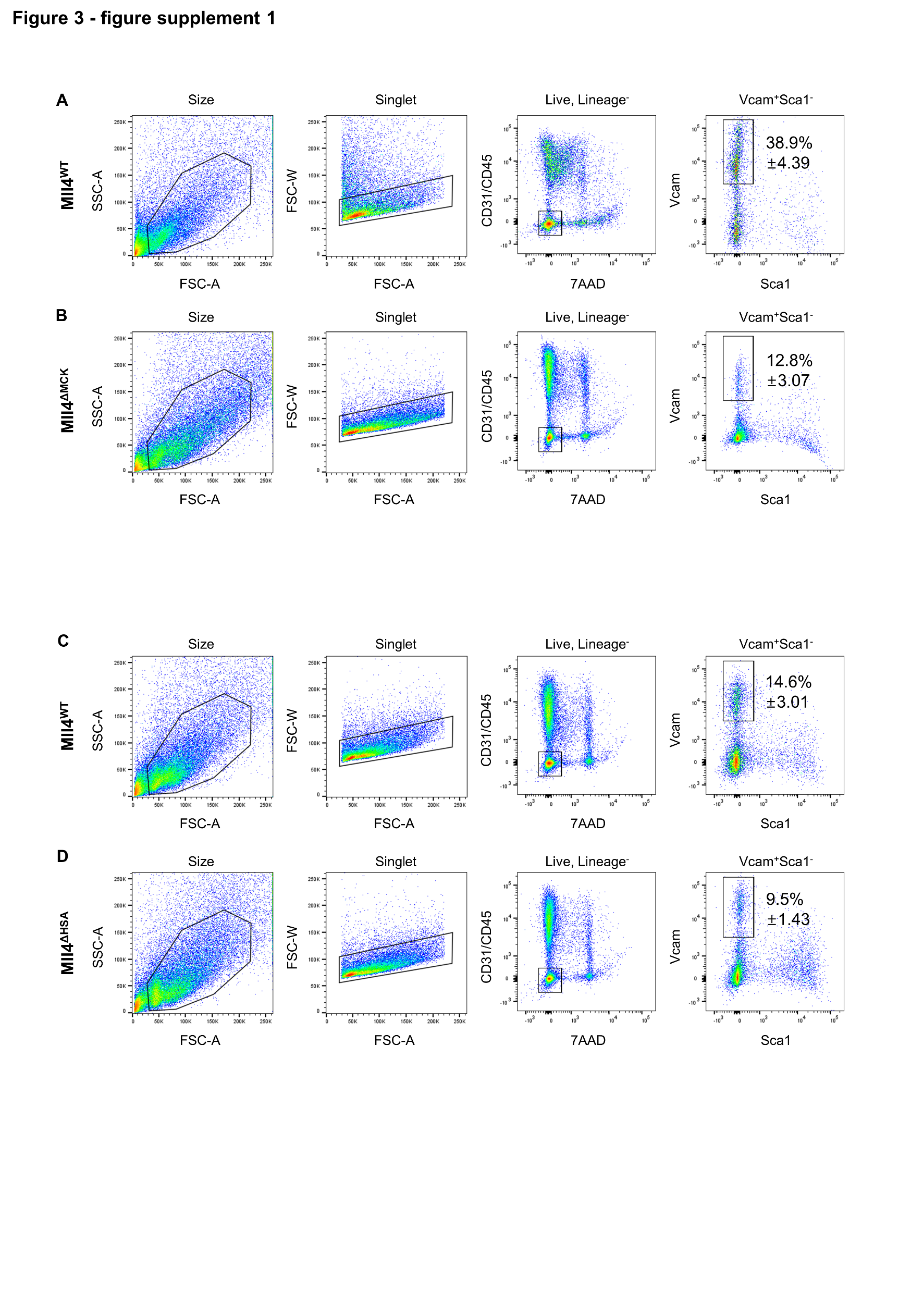


**Supplementary figure 2. Flow cytometry of cell surface markers of limb muscles to isolate MuSCs.**

**(A-D)** Gating strategies for isolating MuSCs in Mll4^ΔMCK^ and Mll4^ΔHSA^ muscles with control Mll4^WT^ muscles for each group. Each figure is representative of n = 3 mice. Data are presented as mean ± SEM of biological replicates. Statistical analyses were performed using unpaired t-test with Welch’s correction.

**
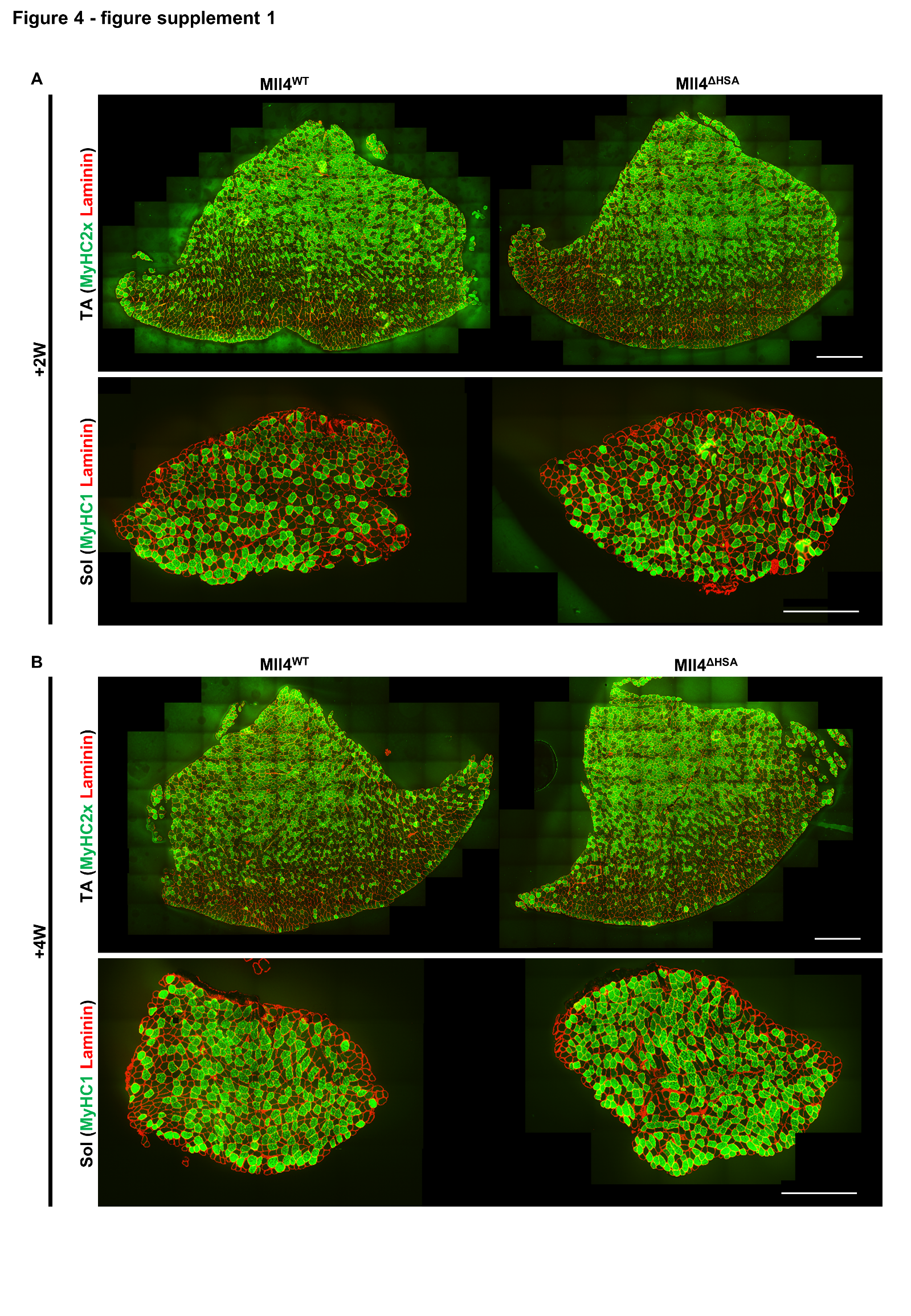
**

**Supplementary figure 3. The fiber area and fiber type composition following Mll4 deletion.**

**(A, B)** Representative image of TA and soleus muscle. TA muscles were labeled with anti-MyHC2x (green) and anti-laminin (red). Soleus muscles were labeled with anti-MyHC1 (green) and anti-laminin (red). Scale bars, 500 μm.


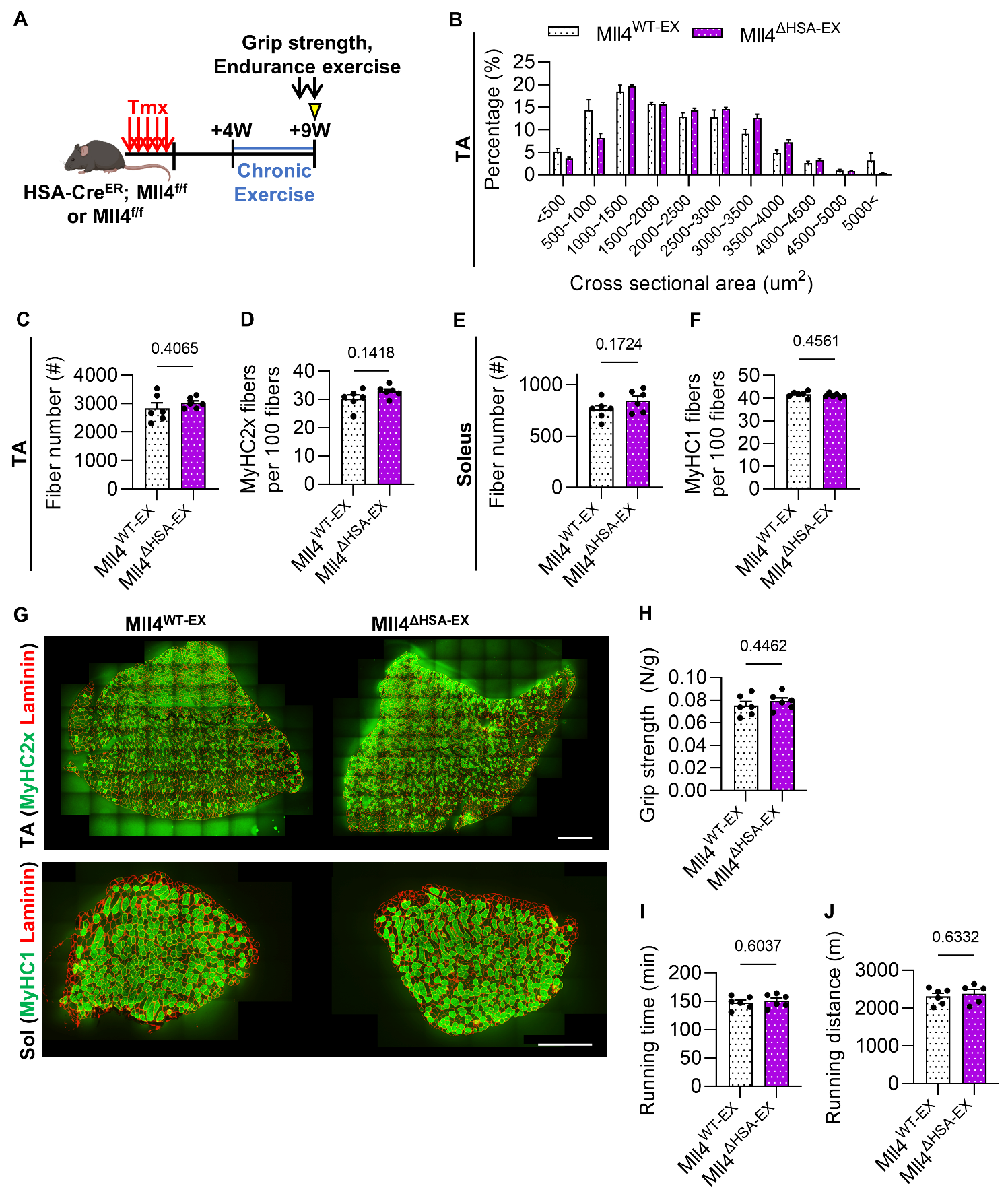


**Supplementary figure 4. Normal exercise capacity and muscle features of myofiber-*Mll4* deleted muscles.**

**(A)** Schematic diagram of mouse training and preparation. **(B)** Percentage of myofibers within each indicated range of CSA, **(C)** gross fiber number, and **(D)** percentage of MyHC2x fibers in TA muscle of Mll4^ΔWT-EX^ and Mll4^ΔHSA-EX^ mice. **(E)** Gross fiber number and **(F)** percentage of MyHC1 fibers in soleus muscle of Mll4^ΔWT-EX^ and Mll4^ΔHSA-EX^ mice. **(G)** Representative image of TA and soleus muscle. TA muscles were labeled with anti-MyHC2x (green) and anti-laminin (red). Soleus muscles were labeled with anti-MyHC1 (green) and anti-laminin (red). Scale bars, 500 μm. **(H)** The measurement values of grip strength (N/g) of Mll4^ΔWT-EX^ and Mll4^ΔHSA-EX^ mice. Grip strength (N) was normalized to body weight (g). **(I, J)** The measurement values of endurance running test. Total running time (min) (I) and total running distance (m) (J) of Mll4^ΔWT-EX^ and Mll4^ΔHSA-EX^ mice. (B-I) n=6 mice for each genotype. Data are presented as mean ± SEM of biological replicates. Statistical analyses were performed using unpaired t-test with Welch’s correction.

**
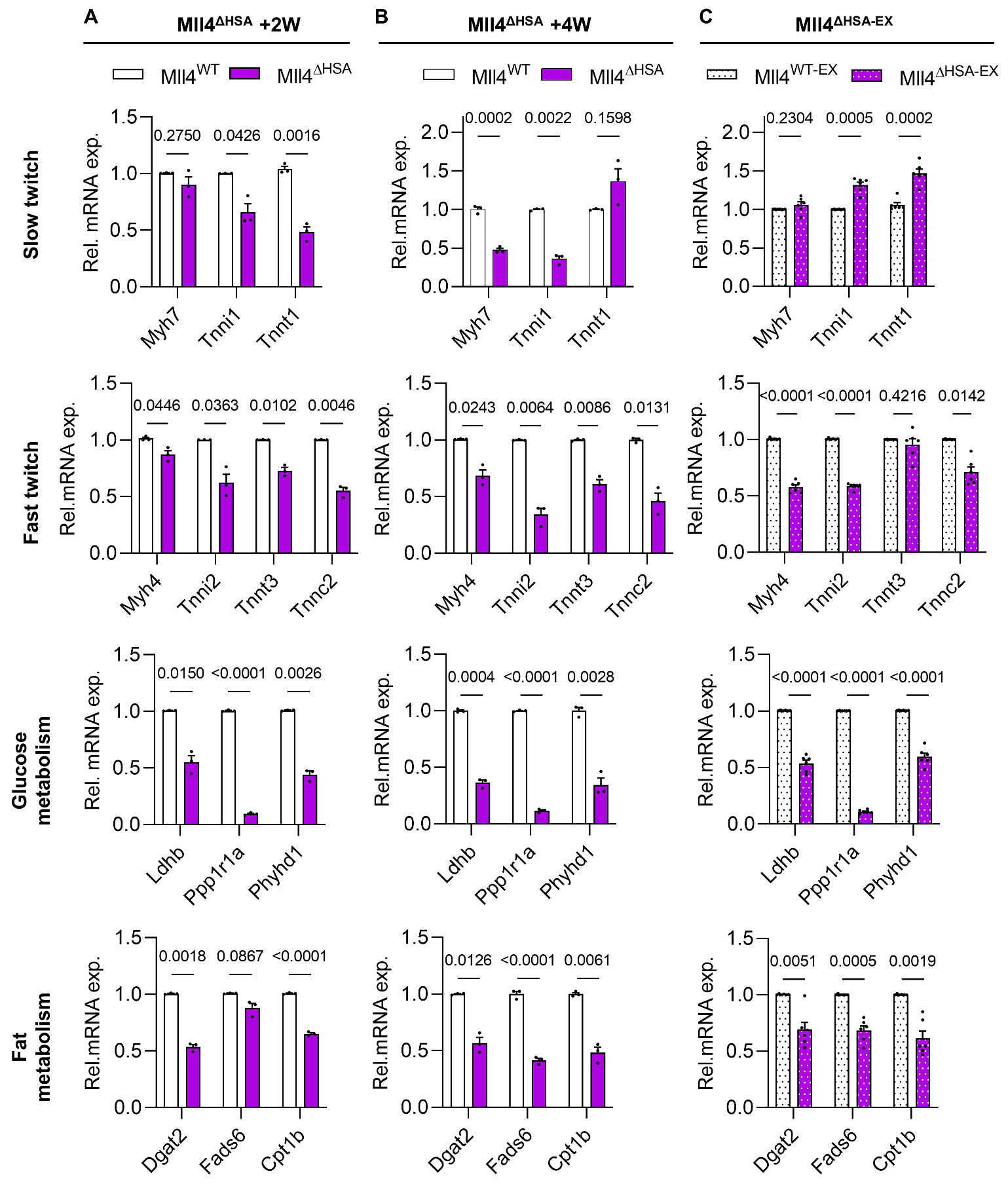
**

**Supplementary figure 5. Gene expression related to fiber type and metabolism in *Mll4*-ablated muscle under various conditions.**

**(A-C)** Normalized expression of representative genes of slow-twitch fiber, fast-twitch fiber, glucose metabolism, and fat metabolism in TA muscle from Mll4^ΔHSA^ +2W (A), Mll4^ΔHSA^ +4W (B), and Mll4^ΔHSA-EX^ (C) mice. (A, B) n=3 mice for each genotype. (C) n=6 mice for each genotype. (A, B, and C) Data are presented as mean ± SEM of biological replicates. Statistical analyses were performed using unpaired t-test with Welch’s correction.


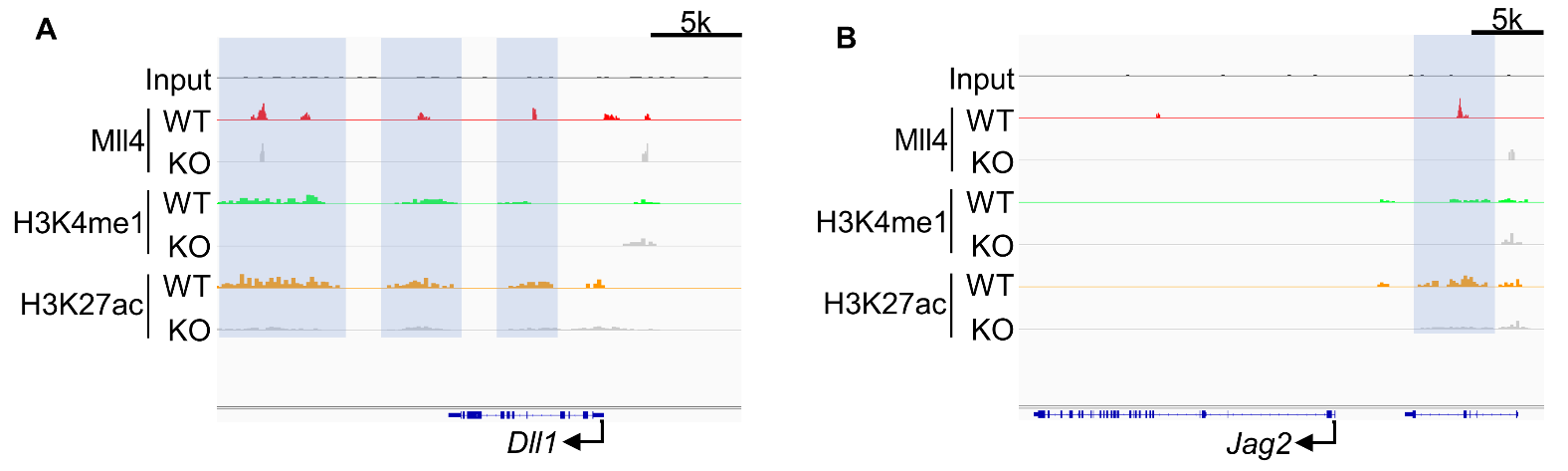


**Supplementary figure 6. ChIP-seq analysis of MLL4 in myocytes**

**(A, B)** Potential binding site of MLL4 to *Dll1* and *Jag2* gene loci.
